# Profiles of Fear of Root Canal Treatment in Adult Endodontic Patients: A Cross-Sectional Study

**DOI:** 10.3390/healthcare14152360

**Published:** 2026-08-03

**Authors:** Mehmet Eren Günner, Öznur Eraslan

**Affiliations:** Department of Endodontics, Faculty of Dentistry, Selçuk University, Akademi Mahallesi, Yeni İstanbul Caddesi No: 309, Selçuklu, 42130 Konya, Türkiye; oznur.eraslan@selcuk.edu.tr

**Keywords:** dental avoidance, endodontic anxiety, fear severity patterns, latent class analysis, preoperative anxiety, root canal treatment fear, trigger-content profiles

## Abstract

**Highlights:**

**What are the main findings?**

- Fear of root canal treatment was heterogeneous; participants showed broad fear severity patterns and exploratory trigger-content profiles.
- The proportion with severe dental anxiety increased descriptively across categories defined by higher numbers of reported fear triggers and sources of negative experiences or exposures.

**What are the implications of the main findings?**

- A brief assessment combining anxiety severity, specific triggers, and avoidance history may help identify patients who need additional support.
- Profile-informed communication and anxiety management strategies are clinically plausible but require prospective evaluation.

**Abstract:**

**Introduction:** Fear of root canal treatment (RCT) may affect care seeking and treatment experience but is often summarized using anxiety scores. This study identified fear severity patterns and trigger-content profiles and examined associations with avoidance and negative experiences. **Materials and Methods:** In this single-center cross-sectional study, 219 of 247 adults scheduled for primary RCT were analyzed after 28 exclusions. Preoperative anxiety was measured using a 0–10 cm Visual Analog Scale for Anxiety (VAS-A). Hierarchical cluster analysis identified fear severity patterns, and latent class analysis identified trigger-content profiles among participants endorsing at least one trigger. **Results:** At least one trigger was endorsed by 75.8%; 21.9% had severe anxiety (VAS-A ≥ 7.0). Severe anxiety increased descriptively from 1.9% with no trigger to 54.3% with three or more triggers. Hierarchical clustering identified low-fear (n = 152) and high-fear (n = 67) patterns. In bivariate comparisons, high-fear membership was more common among women, participants postponing dental visits, and those with negative childhood or adult RCT experiences, and less common among regular dental attenders. Hierarchical multivariable logistic regression showed that female sex and negative adult RCT experience were associated with higher odds of high-fear pattern membership, whereas regular dental attendance was associated with lower odds; negative childhood RCT experience and postponement were not independently significant in the fully adjusted model. Latent class analysis yielded needle-focused (n = 38) and procedural (n = 128) profiles; the latter showed a broader trigger range, while VAS-A distributions were similar. **Conclusions:** Fear of RCT was heterogeneous. Assessing severity, triggers, and avoidance history may support individualized communication. The profiles require external validation before use.

## 1. Introduction

Dental anxiety and dental fear are clinically important psychological variables that affect the timing of dental treatment, tolerance of dental procedures, patient-clinician communication, and subsequent dental attendance [1,2]. In this article, dental anxiety refers to apprehension related to the dental setting or an anticipated dental procedure, whereas dental fear refers to a response to a more identifiable stimulus, such as injections, pain, instrument sounds, or a specific dental treatment [2,3]. The term dental phobia is reserved for a more severe diagnostic construct requiring formal clinical criteria, and no diagnosis of dental phobia was made in this study [3,4].

Dental fear and anxiety are common, but their severity and clinical consequences vary between individuals. A systematic review and meta-analysis estimated the global prevalence of dental fear in adults at 15.3%, high dental fear and anxiety at 12.4%, and severe dental fear and anxiety at 3.3% [5]. Dental fear has been associated with female sex, younger age, previous negative dental experiences, psychological distress, and poorer perceived oral health [2,6]. A recent adult e-survey also linked higher dental anxiety with unpleasant dental experiences, avoidance-related behavior, and adverse emotional reactions in dental settings [7]. Dental fear should therefore be considered not merely a subjective discomfort but a clinical variable that may influence the use of dental services.

The clinical significance of dental anxiety extends beyond transient distress during treatment. High dental fear is often conceptualized within a fear-avoidance cycle, in which fear contributes to the postponement of dental visits, symptom-driven rather than preventive attendance, the accumulation of more complex treatment needs, and the reinforcement of negative dental experiences [8,9]. This cycle may affect oral health-related quality of life and broader health behaviors [10,11]. From this perspective, the clinically relevant question is not only whether a patient is anxious, but also which cues and previous experiences maintain that fear.

Multiple pathways have been proposed for the acquisition and maintenance of dental fear. Direct painful or uncontrollable dental experiences, observation of others’ negative experiences, verbal information from family or the social environment, negative media exposure, and cognitive perceptions of threat or uncontrollability may contribute [9,12,13]. Psychological studies likewise indicate that fear may be acquired through direct conditioning, observational learning, and verbal information [14,15,16]. This framework is particularly relevant to RCT, which patients often perceive as painful, lengthy, invasive, and uncertain [17,18]. Recent endodontic studies indicate that anxiety remains present immediately before RCT and may vary with demographic factors, previous treatment experience, and the broader dental care context [19,20].

Although anticipated pain occupies a central place in patients’ perceptions of RCT, expected and experienced pain do not always correspond [21,22]. Previous studies have shown that dental anxiety may be associated with preoperative and intraoperative pain during endodontic treatment and that patients with higher anxiety may report more intense intraoperative pain [23,24]. However, fear of RCT cannot be adequately characterized by anticipated pain alone. Local anesthetic injection, the sensation of endodontic files, instrument sounds, prolonged mouth opening, pressure applied to a sensitive tooth, postoperative symptoms, and the possibility of treatment failure may also act as endodontic-specific fear triggers [12,25].

Several instruments are used to measure dental anxiety, including the Dental Anxiety Scale, Modified Dental Anxiety Scale, Dental Fear Survey, and visual analog scales [1,26,27]. These instruments are clinically useful; however, total scores may not always identify the specific stimuli that drive an individual patient’s fear [9,25]. The Visual Analog Scale for Anxiety (VAS-A) is a practical single-item measure for preoperative anxiety because it is quick and easy to administer at the chairside [28,29,30]. In this study, the VAS-A was used as a measure of situational preoperative anxiety severity rather than as a diagnostic criterion for dental phobia.

Most studies of endodontic anxiety have examined total anxiety scores, general dental anxiety instruments, or changes in anxiety before and after treatment [23,24,31,32]. Although valuable, this approach may incompletely represent patient-level heterogeneity in RCT fear. Two patients with similar total anxiety scores may fear RCT for different reasons. One patient may be primarily needle-focused, whereas another may fear a combination of pain, prolonged mouth opening, instrument sensations, sounds, and treatment failure. This distinction is important because different fear contents may call for different communication and anxiety management approaches [9,25]. Recent cluster-based research in adults with high dental anxiety further supports the value of examining heterogeneous response patterns rather than relying exclusively on total scores [33].

Person-centered analytic approaches provide an appropriate framework for examining this heterogeneity. Whereas variable-centered analyses estimate relationships among variables, person-centered analyses group individuals with similar response patterns and can identify exploratory subgroups that may be clinically recognizable without implying fixed patient types [33]. In this study, hierarchical clustering and latent class analysis (LCA) were used as complementary methods. Hierarchical clustering addressed the question “How much does the patient fear RCT?”, whereas LCA addressed “Which aspects of RCT does the patient fear?”

The primary aim of this cross-sectional study was to identify exploratory, clinically interpretable RCT fear severity patterns and trigger-content profiles among adults scheduled for primary RCT and to examine the associations of these person-centered classifications with dental attendance, postponement of dental visits, previous negative RCT experiences, and family or social experiences and media exposures. We hypothesized that RCT fear would show heterogeneous fear severity patterns and trigger-content profiles.

## 2. Materials and Methods

This single-center, cross-sectional, structured questionnaire-based observational study was conducted among adult patients who presented to the Department of Endodontics, Faculty of Dentistry, Selçuk University, and were scheduled for primary RCT. Data were collected from 1 April 2025 through 31 March 2026. Reporting was structured in accordance with the STROBE recommendations for cross-sectional observational studies [34].

Eligible patients were enrolled consecutively during the predefined data collection period. The inclusion criteria were presentation to the endodontic clinic; a clinical and radiographic indication for primary RCT; age 18–60 years; ability to read and understand Turkish; ability to complete the questionnaire independently before treatment; and provision of written informed consent. Both symptomatic and asymptomatic patients were eligible, provided that primary RCT was planned. Pulpal and apical diagnoses, the presence or intensity of acute pain, analgesic use, and other preoperative clinical characteristics were not predefined study variables and were not systematically recorded for the present analyses.

Before questionnaire administration, patients were assessed for eligibility. Patients scheduled for retreatment of a previously root-filled tooth, those outside the predefined age range, those with cognitive or communication limitations preventing independent questionnaire completion, those who had previously participated in the study, and those presenting for a procedure other than primary RCT were not enrolled and did not receive the questionnaire. The recruitment count was restricted to patients scheduled for primary RCT; other patients treated in the clinic were outside the study population.

During the study period, 425 patients scheduled for primary RCT were invited to participate. Of these, 247 (58.1%) provided written informed consent and completed the questionnaire, whereas 178 declined participation. Questionnaires were excluded from the analysis if they contained missing responses required for the principal analyses or internally inconsistent responses. Six questionnaires were incomplete and 22 contained internally inconsistent responses. Internal inconsistency criteria were defined before final analytical coding as mutually incompatible answers to logically related items. The principal rules were reporting no previous RCT experience while evaluating a previous RCT experience and providing incompatible responses in conditional follow-up items concerning previous negative experiences or exposures. Because a single questionnaire could contain more than one inconsistency, exclusions were recorded at the questionnaire level rather than as mutually exclusive rule-specific counts. Thus, 28 questionnaires met the data quality exclusion criteria, resulting in a final analytical sample of 219 participants. No values were imputed. After questionnaire-level exclusions, the variables required for the primary hierarchical cluster analysis and multivariable logistic regression were complete, and both analyses included all 219 participants.

Sample size was determined a priori for selected chi-square comparisons using a medium effect size (Cohen’s w = 0.25), α = 0.05, and 90% power, yielding a minimum required sample size of 203 participants. The final sample comprised 219 participants. This calculation was intended only for selected categorical comparisons and was not used to establish the adequacy, stability, or reproducibility of the hierarchical clustering, latent class analysis, or multivariable regression models, which should be regarded as exploratory. The analysis was performed using G*Power 3.1.9.7.

All participants received verbal and written information about the study and provided written informed consent before participation. Personal data were processed anonymously and used solely for scientific research. The study was approved at the meeting of the Non-Interventional Clinical Research Evaluation Committee of the Faculty of Dentistry, Selçuk University, held on 10 March 2025 (approval no. 2025/26).

Participants who met the eligibility criteria completed a structured questionnaire before the RCT. The questionnaire was provided by a study investigator before treatment and completed without guidance from the clinician who would perform the procedure. Only technical instructions on how to mark responses were given; no explanation that could influence response content was provided. This procedure was used to reduce bias related to interviewer and clinician influence. Consecutive enrollment was intended to reduce discretionary selection during recruitment; potential non-participation, recall, and residual information biases were considered in the interpretation and are addressed in the limitations.

The questionnaire recorded sex, age group, educational level, self-reported regular dental attendance, postponement of dental visits, reasons for postponement, the dental procedure perceived as most frightening, fear when thinking about visiting the dentist, fear when sitting in the dental chair, previous RCT experience, RCT fear triggers, negative RCT experiences in childhood and adulthood, negative RCT experiences among family members or in the social environment, negative media exposure related to RCT, and preoperative anxiety.

The questionnaire content was developed from items and concepts used in the literature on general dental anxiety and endodontic fear. Items concerning thinking about visiting the dentist and sitting in the dental chair were consistent with the anticipatory and clinical setting components emphasized in the DAS/MDAS tradition [1,27]. Endodontic-specific triggers were adapted from the Endodontic Fear Survey and Dental Fear Survey and included local anesthetic injection, fear of pain during treatment, postoperative pain, postoperative swelling, instrument sounds, the sensation of endodontic instruments, prolonged mouth opening, and the possibility of treatment failure [25,26]. This study was not designed as a scale development or psychometric validation study. Rather, the questionnaire was structured as a multidimensional descriptive tool to capture clinically relevant domains of RCT fear and to support fear profiling using items and concepts drawn from established dental anxiety and endodontic fear instruments.

Preoperative anxiety was assessed immediately before treatment using the Visual Analog Scale for Anxiety (VAS-A). Participants marked their current preoperative anxiety level on a 0–10 cm visual analog line, and the marked distance was recorded in centimeters. VAS-A scores were classified according to the cutoffs proposed by Facco et al. [29]: 0–5.0 cm, no/low dental anxiety; 5.1–6.9 cm, dental anxiety; and ≥7.0 cm, severe dental anxiety.

Sociodemographic variables comprised sex, age group, and educational level. Dental behavior variables included self-reported regular dental attendance, postponement of dental visits, and reasons for postponement. RCT-specific variables comprised previous RCT, endorsement of at least one RCT fear trigger, and specific fear triggers. Previous RCT experience was coded as a binary variable and did not capture its timing, frequency, perceived quality, pain intensity, or treatment outcome. The binary variable “endorsement of at least one RCT fear trigger” indicated whether one or more RCT fear triggers were coded as endorsed. Fear triggers were coded as dichotomous multiple-response items that were not mutually exclusive: injection/needle, fear of pain during treatment, fear of postoperative pain, fear of postoperative swelling, instrument sounds, sensation of endodontic instruments, prolonged mouth opening, and possibility of treatment failure. Accordingly, trigger percentages could exceed 100% in total, and the independent or relative contribution of individual triggers could not be inferred from endorsement frequencies. Sources of negative experiences or exposures were summarized as negative RCT experience in childhood, negative RCT experience in adulthood, negative RCT experience among family members or within the social environment, and negative media exposure related to RCT. The cumulative number of negative sources was calculated by summing these sources.

### Statistical Procedures

Categorical variables were summarized as frequencies and percentages. Relationships between categorical variables were examined using cross-tabulations. Pearson’s chi-square test was used when assumptions were met, and Fisher’s exact test was used for sparse contingency tables or when expected cell counts were insufficient. The significance threshold was set at *p* < 0.05. Cramer’s V was reported as an effect size measure for selected chi-square tests. Bivariate analyses were interpreted descriptively and were not intended to establish causal relationships. Statistical analyses were performed using IBM SPSS Statistics 24.0, jamovi 2.4, Microsoft Excel 2016, and RStudio 2023.09.0. Because the secondary comparisons were exploratory, no multiplicity adjustment was applied; exact *p*-values and effect sizes were reported and interpreted cautiously. No sampling weights were applied because participants were enrolled consecutively from a single clinic rather than through a complex sampling design.

Fear severity patterns were identified using hierarchical cluster analysis. The primary analysis used five nonredundant indicators: fear when thinking about visiting the dentist (none, low, moderate, high, or very high), fear when sitting in the dental chair (none, low, moderate, high, or very high), number of endorsed RCT fear triggers (no trigger, one trigger, two triggers, or three or more triggers), fear at the prospect of undergoing RCT (none, low, moderate, high, or very high), and VAS-A classification (no/low dental anxiety, dental anxiety, or severe dental anxiety). The binary indicator for endorsement of at least one RCT fear trigger was excluded because it was deterministically derived from the trigger-count variable. All indicators were coded in the same direction, with higher values indicating greater fear or anxiety, and were z-standardized before the primary clustering analysis. Euclidean distance and Ward’s method were used for the primary analysis [35]. Two- and three-cluster solutions were compared using the average silhouette coefficient, cluster size, parsimony, and clinical interpretability. As a robustness analysis appropriate for mixed and ordinal data, hierarchical clustering was repeated using Gower distance with average linkage. Participant-level agreement between the Ward–Euclidean and Gower-based two-cluster solutions was quantified using the adjusted Rand index, overall and cluster-specific Jaccard coefficients, and the proportion of participants assigned to corresponding clusters. The resulting labels were descriptive and were not intended as diagnostic categories. Comparisons involving indicators used to derive the patterns were interpreted descriptively rather than as independent confirmatory tests. Additional details on indicator coding, internal validation, robustness analysis, and participant-level agreement are provided in Supplementary Tables S1 and S2.

To examine whether selected external characteristics were associated with high-fear pattern membership after adjustment, hierarchical multivariable binary logistic regression analyses were performed with high-fear pattern membership as the dependent variable. To make the conceptual assumptions and predictor-domain structure transparent, a directed acyclic graph was developed (Figure S1). The graph distinguishes background characteristics, previous negative RCT experiences, dental attendance and avoidance history, and high-fear pattern membership. Arrows represent prespecified hypothesized temporal or structural relationships rather than empirically established causal effects because all variables were assessed cross-sectionally. For the hierarchical association analysis, predictors were entered in prespecified blocks: background characteristics (sex and age group) in Model 1; regular dental attendance and postponement of dental visits added in Model 2; and previous negative RCT experiences during childhood and adulthood added in Model 3. This sequence was used to examine the stability of adjusted associations as covariate domains were added and was not interpreted as a causal or mediational sequence. Variables used to define the fear severity patterns, including the number of RCT fear triggers, were not entered as predictors. Adjusted odds ratios (AORs), 95% confidence intervals (CIs), and *p*-values were reported for each model.

Overall model performance was evaluated using the likelihood-ratio chi-square test, Akaike information criterion (AIC), Bayesian information criterion (BIC), McFadden’s and Cox–Snell pseudo-R^2^ values, and the Hosmer–Lemeshow goodness-of-fit test. Multicollinearity was assessed using variance inflation factors and tolerance statistics, whereas potential sparse-data instability and separation were evaluated by examining model convergence, standard errors, and confidence intervals. Predictor selection was based on the study’s conceptual framework and clinical relevance rather than on univariable statistical significance, and no data-driven variable selection procedures were applied. All 219 participants were included in the analyses. No interaction terms were fitted because the exploratory sample size and sparse categories did not support reliable effect-modification estimates. The regression analyses were interpreted as adjusted association analyses rather than evidence of causality. Pathway or mediation analyses were not performed because all variables were measured contemporaneously before treatment, precluding the establishment of temporal ordering required for causal pathway modeling.

Exploratory trigger-content profiles were examined using LCA among participants who endorsed at least one RCT fear trigger (n = 166). Eight dichotomous fear triggers were included. Two- and three-class models were compared using log-likelihood, Akaike information criterion (AIC), Bayesian information criterion (BIC), entropy, estimated class proportions, class size, and clinical interpretability. Each model was estimated using 100 random starting values, a maximum of 5000 iterations, and a convergence tolerance of 1 × 10^−8^. As an additional convergence check, the retained two-class model was re-estimated independently five times using 100 random starts per run; all runs converged to the same maximum log-likelihood (−626.81). BIC was treated as the primary parsimony criterion, while AIC, entropy, class size, and clinical coherence were considered jointly as secondary criteria. The two-class solution was retained because of its lower BIC, parsimony, and clinical interpretability, while the three-class solution was considered a plausible exploratory alternative. Participants were assigned to the class with the highest posterior membership probability for subsequent descriptive cross-tabulations; model-estimated class proportions and modal assignment counts were reported separately. Average posterior probabilities, mean maximum posterior probability, classification uncertainty, and the proportion of participants with maximum posterior probability below 0.70 were used to describe classification quality. Local independence was assessed using bivariate residuals across all 28 item pairs. External comparisons based on modal class assignment were treated as descriptive because they did not incorporate classification error and may therefore have been attenuated or distorted by assignment uncertainty. Class labels were descriptive and were not intended to represent diagnostic categories or stable clinical phenotypes. Fear of pain during RCT was interpreted as a fear trigger rather than as a measure of actual pain intensity. Detailed model-fit results, class-specific item-response probabilities, the alternative three-class solution, and classification diagnostics are provided in Supplementary Tables S3–S6.

## 3. Results

### 3.1. Participant Characteristics and Dental Attendance

**Figure 1:**
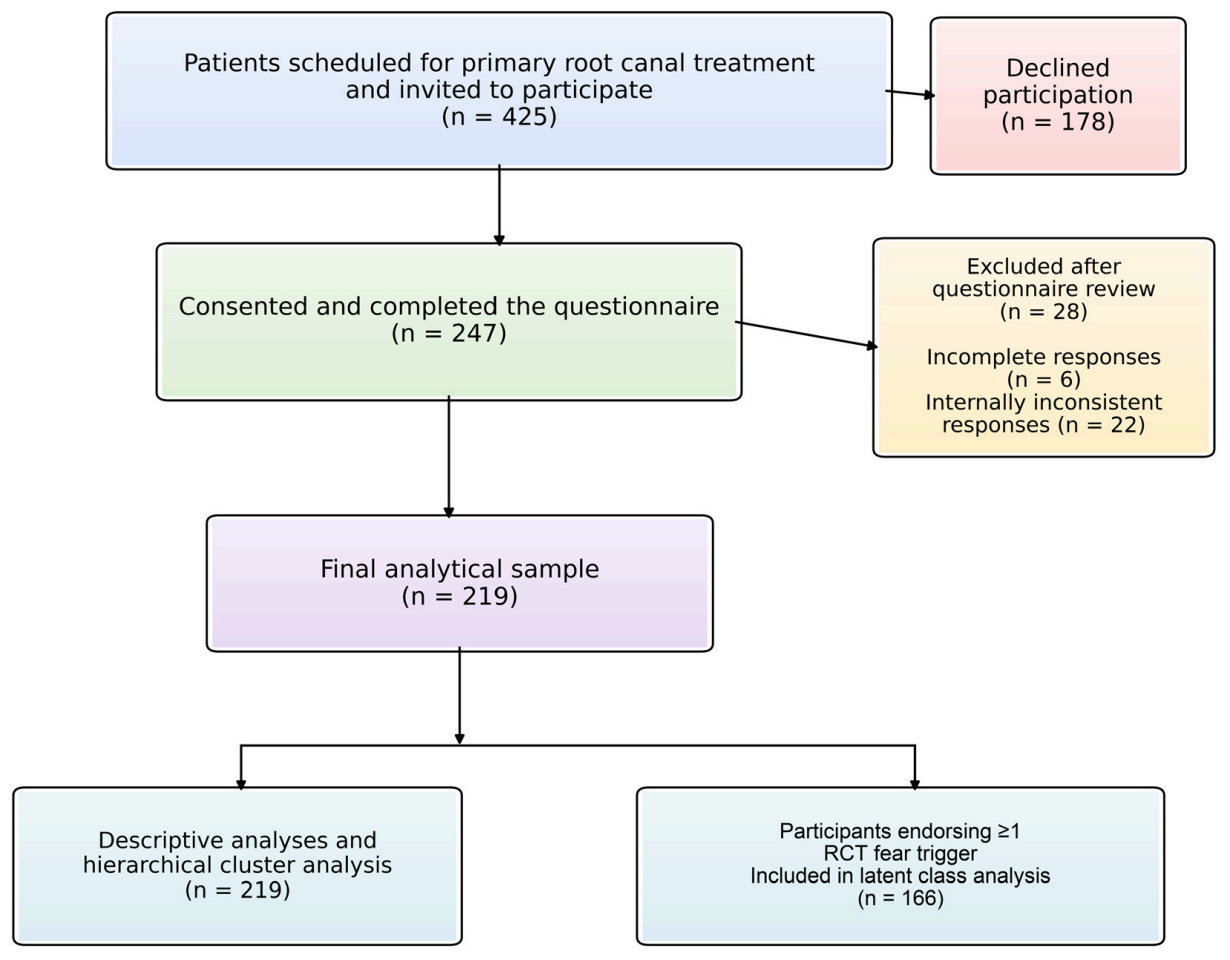
Flow of participants through recruitment and analysis. Descriptive analyses and hierarchical cluster analysis included the final analytical sample (n = 219); latent class analysis was restricted to participants who endorsed at least one RCT fear trigger (n = 166).

Among 425 patients scheduled for primary RCT and invited to participate, 247 (58.1%) consented and completed the questionnaire, whereas 178 declined participation. Of the 247 questionnaires, 6 were excluded for incomplete responses and 22 for internally inconsistent responses, resulting in a final analytical sample of 219 participants. The sex distribution was balanced: 109 participants were women (49.8%) and 110 were men (50.2%). The largest age groups were 26–35 years (28.3%) and 18–25 years (25.6%). Most participants had a high school or university-level education. The participant flow is shown in Figure 1.

**Figure 2:**
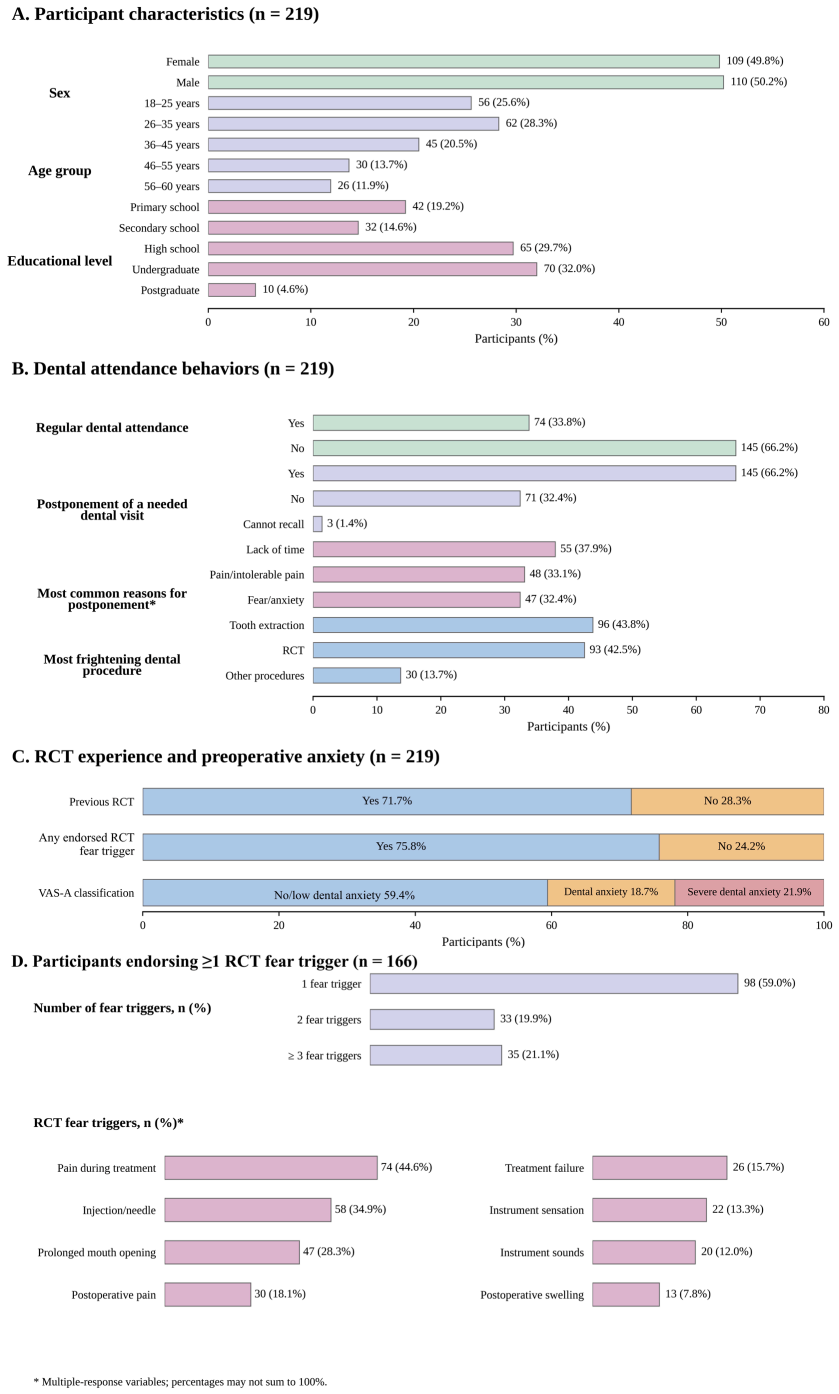
Participant characteristics, dental attendance behaviors, RCT experience, preoperative anxiety, and fear triggers. Panels (**A**–**D**) present participant characteristics, dental attendance behaviors, RCT experience and preoperative anxiety, and the number and distribution of fear triggers among participants who endorsed at least one RCT fear trigger, respectively. Values are presented as n (%) or percentages. RCT, root canal treatment; VAS-A, Visual Analog Scale for Anxiety. * Multiple responses were permitted. Only the three most frequently reported reasons for postponement are shown, whereas all eight RCT fear triggers are displayed. Percentages for postponement reasons were calculated among participants who reported postponement (n = 145), and percentages for RCT fear triggers were calculated among participants who endorsed at least one RCT fear trigger (n = 166).

Only 33.8% of participants reported regular dental attendance, whereas 66.2% reported that they did not attend regularly. Similarly, 66.2% reported postponing a dental visit despite knowing it was necessary. The most common reasons for postponement were lack of time, absence of pain or tolerable pain, and fear/anxiety. Tooth extraction (43.8%) and RCT (42.5%) were reported at nearly equal rates as the most frightening dental procedures. Participant characteristics, dental attendance behaviors, and key RCT fear variables are summarized in Figure 2.

Among the 166 participants who endorsed at least one RCT fear trigger, the most frequently reported trigger was fear of pain during treatment (n = 74, 44.6%), followed by injection/needle fear (n = 58, 34.9%), prolonged mouth opening (n = 47, 28.3%), postoperative pain (n = 30, 18.1%), the possibility of treatment failure (n = 26, 15.7%), sensation of endodontic instruments (n = 22, 13.3%), instrument sounds (n = 20, 12.0%), and postoperative swelling (n = 13, 7.8%). Multiple responses were permitted, and the triggers were not mutually exclusive; these frequencies therefore describe endorsement patterns and do not estimate the independent contribution of each trigger to overall fear.

The proportion of participants endorsing at least one RCT fear trigger was similar among participants with and without previous RCT experience (76.4% vs. 74.2%; *p* = 0.727). Because individual triggers were recorded as multiple-response items that were not mutually exclusive, trigger frequencies according to previous RCT experience are presented descriptively. Among participants without previous RCT experience, 40.3% reported fear of pain during treatment and 19.4% reported fear of postoperative pain, compared with 31.2% and 11.5%, respectively, among those with previous RCT experience. Conversely, instrument sounds and prolonged mouth opening were reported by 12.1% and 24.8%, respectively, of participants with previous RCT experience, compared with 1.6% and 12.9% of those without previous RCT experience.

### 3.2. RCT Fear Burden, VAS-A Classification, and Sources of Negative Experiences or Exposures

According to the VAS-A classification, 130 participants (59.4%) had no/low dental anxiety, 41 (18.7%) had dental anxiety, and 48 (21.9%) had severe dental anxiety. Overall, 40.6% of participants were classified as having dental anxiety or severe dental anxiety.

Severe dental anxiety was identified in 35.8% of women (39/109) and 8.2% of men (9/110) (χ^2^ = 31.05, *p* < 0.001).

The distribution of VAS-A classifications differed across RCT fear-trigger count categories. Severe dental anxiety was present in 1.9% of participants who reported no RCT fear trigger, 18.4% of those reporting one trigger, 30.3% of those reporting two triggers, and 54.3% of those reporting three or more triggers (χ^2^ = 69.21, *p* < 0.001; Cramer’s V = 0.40). These proportions increased descriptively across categories; no formal trend model was fitted.

**Figure 3:**
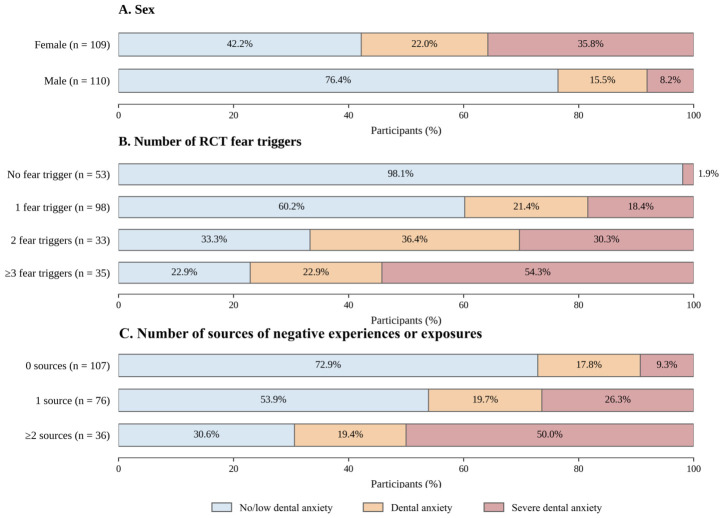
Distribution of preoperative VAS-A classifications by sex, number of RCT fear triggers, and number of sources of negative experiences or exposures. Values are presented as percentages; totals may not sum to 100% because of rounding. RCT, root canal treatment; VAS-A, Visual Analog Scale for Anxiety.

The distribution of VAS-A classifications also differed across categories defined by the number of sources of negative experiences or exposures. Severe dental anxiety was identified in 9.3% of participants who reported no negative source, 26.3% of those who reported one source, and 50.0% of those who reported two or more sources (χ^2^ = 30.15, *p* < 0.001; Cramer’s V = 0.26). These proportions increased descriptively across categories; no formal trend model was fitted. VAS-A classification according to sex, number of RCT fear triggers, and number of sources of negative experiences or exposures is presented in Figure 3.

A negative RCT experience in childhood was reported by 28 participants (12.8%), a negative RCT experience in adulthood by 54 (24.7%), a negative RCT experience among family members or in the social environment by 50 (22.8%), and negative media exposure by 23 (10.5%). Among participants who reported the respective source, the proportions who stated that the corresponding experience or exposure contributed to their current RCT fear were 57.1% (16/28), 61.1% (33/54), 54.0% (27/50), and 78.3% (18/23), respectively.

### 3.3. Fear Severity Patterns

When fear when thinking about visiting the dentist, fear when sitting in the dental chair, the number of RCT fear triggers, fear at the prospect of undergoing RCT, and preoperative anxiety were considered together, participants formed two broad fear severity patterns. The low-fear pattern comprised 152 participants, among whom fear related to the dental setting and RCT generally remained low and the number of reported RCT fear triggers was limited. The high-fear pattern comprised 67 participants, among whom fear was more pronounced across the assessments, from thinking about visiting the dentist and sitting in the dental chair to RCT-specific triggers.

The retained two-cluster solution was compared with an alternative three-cluster solution using the average silhouette coefficient. The mean silhouette coefficient was 0.445 for the two-cluster solution and 0.390 for the three-cluster solution, whose cluster sizes were 124, 28, and 67 participants. The two-cluster solution was retained because it showed better overall cluster quality based on the average silhouette coefficient and provided a parsimonious and clinically interpretable lower- versus higher-fear distinction.

As a robustness analysis, hierarchical clustering was repeated using Gower distance with average linkage, which accommodates the ordinal and count nature of the indicators. The Gower-based two-cluster solution had an average silhouette coefficient of 0.569 and yielded clusters of 172 and 47 participants. Thus, the broad lower- versus higher-fear structure was retained, although exact membership and cluster sizes were partly sensitive to the distance measure and clustering algorithm. Participant-level agreement between the Ward–Euclidean and Gower-based solutions was moderate to substantial (adjusted Rand index = 0.619; overall Jaccard coefficient = 0.744; cluster-specific Jaccard coefficients = 0.873 and 0.676), and 197 of 219 participants (90.0%) were assigned to corresponding clusters.

**Figure 4:**
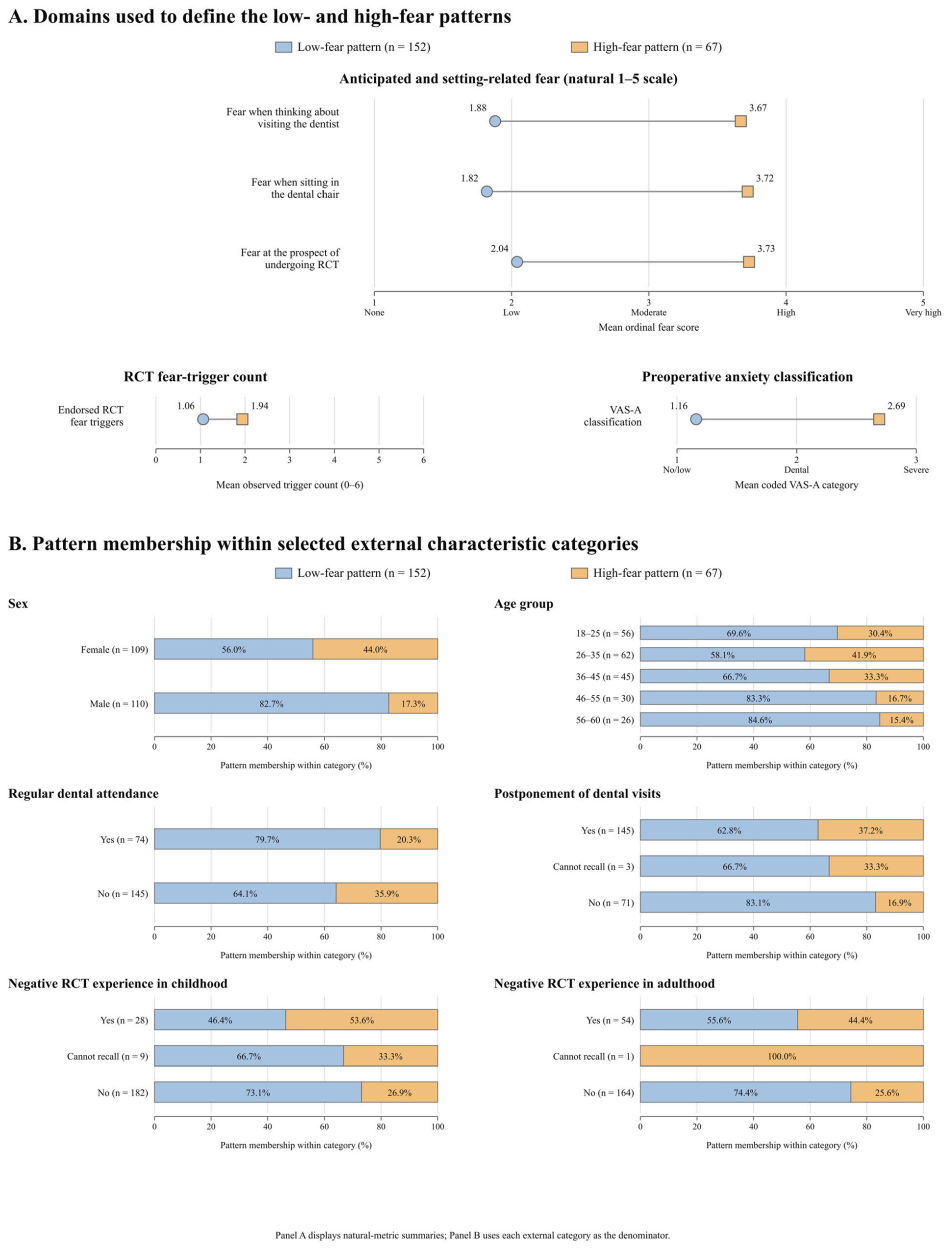
Natural-metric summaries of the pattern-defining domains and pattern membership within selected external-characteristic categories. Note: Panel (**A**) presents natural-metric summaries of the five domains used in the hierarchical cluster analysis. Blue cricles denote the low-fear patternd (n = 152), whereas orange squares denote the high-fear pattern (n = 67). The three ordinal fear indicators were coded from 1 (none) to 5 (very high). For clinical interpretability, the trigger-count domain is displayed as the mean observed number of endorsed RCT fear triggers (0–6); in the hierarchical cluster analysis, it was entered as a four-category indicator (no trigger, one trigger, two triggers, or three or more triggers). VAS-A category was coded as 1 (no/low dental anxiety), 2 (dental anxiety), or 3 (severe dental anxiety). Panel (**B**) presents the distribution of pattern membership within each external-characteristic category. RCT, root canal treatment; VAS-A, Visual Analog Scale for Anxiety.

Selected external characteristics differed descriptively between the fear severity patterns. Women constituted 71.6% of the high-fear pattern compared with 40.1% of the low-fear pattern (*p* < 0.001), and regular dental attendance was less common in the high-fear pattern (22.4% vs. 38.8%; *p* = 0.018). Postponement of dental visits was more common in the high-fear pattern (80.6% vs. 59.9%; *p* = 0.010). Negative RCT experiences in childhood (22.4% vs. 8.6%; *p* = 0.017) and adulthood (35.8% vs. 19.7%; *p* = 0.011) were also more common in the high-fear pattern. The overall age-group comparison was at the conventional significance boundary (χ^2^ = 9.49, *p* = 0.050) and was treated as exploratory. In the high-fear pattern, the 26–35- and 36–45-year age groups accounted for 38.8% and 22.4%, respectively, whereas the 46–55- and 56–60-year groups accounted for 7.5% and 6.0%. The pattern-defining indicators and selected external characteristics of the fear severity patterns are shown in Figure 4.

Hierarchical multivariable binary logistic regression analyses were performed to examine external characteristics associated with high-fear pattern membership (Table 1). Across the three models, model fit improved with the sequential addition of predictor blocks (Supplementary Table S7). The fully adjusted model showed satisfactory overall fit and no problematic multicollinearity, with Model 3 variance inflation factors ranging from 1.025 to 1.055 and tolerance values ranging from 0.948 to 0.975 (Supplementary Table S8).

Female sex remained consistently associated with higher odds of high-fear pattern membership across all three models. In the fully adjusted model, female participants had approximately fourfold higher odds of high-fear pattern membership than male participants (AOR = 4.27, 95% CI: 2.14–8.51, *p* < 0.001). Regular dental attendance was associated with lower odds of high-fear pattern membership (AOR = 0.40, 95% CI: 0.18–0.85, *p* = 0.017). A negative RCT experience in adulthood was associated with higher odds of high-fear pattern membership (AOR = 2.19, 95% CI: 1.00–4.77, *p* = 0.049). In contrast, the association with a negative childhood RCT experience did not reach statistical significance in the fully adjusted model (AOR = 2.52, 95% CI: 0.94–6.72, *p* = 0.066). Postponement of dental visits showed a positive but non-significant association (AOR = 2.08, 95% CI: 0.94–4.60, *p* = 0.070), while age group was not independently associated with high-fear pattern membership.

All 219 participants were included in the analyses. The ‘cannot recall’ category for negative RCT experience in adulthood contained only one participant and yielded a non-informative boundary estimate; its category-specific AOR, 95% CI, and *p*-value were therefore reported as not estimable and were not interpreted.

### 3.4. RCT Trigger-Content Profiles

Trigger-content profile analysis was conducted among 166 participants who endorsed at least one RCT fear trigger. The low- and high-fear patterns described in Section 3.3 represent fear severity, whereas the latent classes in this section represent fear content. Model-selection indices gave conflicting signals. The three-class solution had a lower AIC than the two-class solution (1273.54 vs. 1287.62) and higher entropy (0.948 vs. 0.833), whereas the two-class solution had a lower BIC (1340.53 vs. 1354.45; ΔBIC = 13.92 in favor of the two-class model). The alternative three-class solution comprised estimated classes of 64.5%, 12.4%, and 23.2%. Its item-response probabilities suggested a lower-probability procedural class, a needle-focused class, and a broader pain/procedural class, respectively. Although the two-class solution was retained because of its lower BIC, greater parsimony, and clinical interpretability, the three-class solution remains a plausible alternative representation of the data. The retained solution should be regarded as a parsimonious, exploratory modeling choice rather than evidence that only two latent classes can exist. Panel A of Table 2 presents the model comparison.

In the retained model, the estimated class proportions were 17.7% and 82.3%, whereas classification by maximum posterior probability yielded 38 (22.9%) and 128 (77.1%) participants. This difference is expected because estimated proportions are probabilistic model parameters, whereas modal assignment assigns each participant to the single class with the highest posterior probability. Modal assignments were used for the external comparisons reported in Section 3.5.

Classification quality was high overall but uneven across the two latent classes. Average posterior probabilities were 0.766 for the needle-focused profile, indicating moderate classification certainty, and 0.998 for the procedural profile, indicating very high certainty. The mean maximum posterior probability was 0.945, mean classification uncertainty was 0.055, and 6.0% of participants had a maximum posterior probability below 0.70. Six of the 28 bivariate residuals exceeded 3.84, although none exceeded 10.0. Accordingly, the local-independence assumption should be interpreted with caution.

Under modal assignment, the smaller class comprised 38 participants and was labeled the needle-focused profile because endorsement was concentrated on injection/needle fear. The model estimated an item-response probability of 1.000 for injection/needle fear, compared with 0.147 for fear of pain during treatment and 0.046 for postoperative pain; the remaining procedural triggers had boundary estimates of 0.000. The larger class comprised 128 participants and was labeled the procedural profile because endorsement extended across several stages of RCT. Its highest item-response probabilities were fear of pain during treatment (0.510), prolonged mouth opening (0.344), injection/needle fear (0.217), postoperative pain (0.210), and treatment failure (0.190), with lower probabilities for instrument sensation, instrument sounds, and postoperative swelling. The labels summarize relative response patterns rather than fixed clinical phenotypes. Panel B of Table 2 presents the item-response probabilities for the retained two-class solution. Under modal assignment, most participants in both profiles endorsed one trigger: 28 of 38 participants (73.7%) in the needle-focused profile and 70 of 128 (54.7%) in the procedural profile. Multiple triggers were more common in the procedural profile (45.3% vs. 26.3%), and endorsement of three to six triggers occurred only in that profile. Thus, the broader trigger range of the procedural profile represents a class-level response pattern rather than evidence that most members simultaneously feared multiple stages of treatment.

### 3.5. Clinical and Experiential Characteristics of the Trigger-Content Profiles

To determine whether the two trigger-content profiles differed beyond the trigger patterns used to define them, external characteristics were examined in three conceptually distinct domains: the clinical expression of RCT fear, negative RCT experiences, and indirect experiences or exposures. The comparisons in this section were restricted to variables that describe the clinical or experiential meaning of the profiles. Because these comparisons were based on modal class assignment rather than classification-error-adjusted methods, they were considered descriptive and may have been attenuated or distorted by assignment error.

The needle-focused and procedural profiles showed similar overall clinical expression. The distributions of fear at the prospect of undergoing RCT and VAS-A classification did not differ between the profiles (*p* = 0.915 and *p* = 0.693, respectively). Severe dental anxiety was observed in 23.7% of the needle-focused profile and 29.7% of the procedural profile.

Among negative experiences, a negative RCT experience in childhood was more common in the procedural profile than in the needle-focused profile (17.2% vs. 2.6%; *p* = 0.042). This comparison was based on only one affirmative response in the needle-focused profile and should be regarded as exploratory rather than as evidence of a stable or reproducible profile difference. Negative RCT experiences in adulthood did not differ between the profiles (*p* = 0.128). Likewise, the distributions of negative RCT experiences among family members or within the social environment and negative media exposure were comparable (*p* = 0.379 and *p* = 0.245, respectively). Overall, the profiles were distinguished primarily by the content of fear rather than by its overall intensity or by most measured experiential characteristics. The complete comparisons are presented in Table 3.

## 4. Discussion

Fear of RCT cannot be adequately characterized by a single severity indicator. Hierarchical clustering identified broad low- and high-fear patterns, whereas LCA identified exploratory needle-focused and procedural trigger-content profiles. In bivariate comparisons, the high-fear pattern included more women, fewer participants reporting regular dental attendance, more participants who postponed dental visits, and more participants reporting negative RCT experiences in childhood or adulthood. In the adjusted model, female sex and negative RCT experience in adulthood were associated with higher odds of high-fear pattern membership, whereas regular dental attendance was associated with lower odds; negative childhood RCT experience and postponement were not independently significant. Taken together, these findings suggest that RCT fear encompasses related but non-interchangeable dimensions of severity, content, experience, and avoidance behavior.

These findings broadly support the working hypothesis that RCT fear is heterogeneous and can form interpretable fear severity patterns and trigger-content profiles. Recent cluster-based research has likewise identified more than one subtype among adults with high dental anxiety, supporting the usefulness of person-centered approaches while demonstrating that subgroup boundaries depend on the variables and population studied [33]. The revised nonredundant HCA favored a two-pattern solution over the alternative three-pattern solution based on the average silhouette coefficient. The Gower-based analysis recovered the same broad lower- versus higher-fear structure, but the adjusted Rand index, Jaccard coefficients, and different cluster sizes showed that exact membership was partly sensitive to the distance measure and clustering algorithm. The retained fear severity patterns should therefore be interpreted as exploratory broad groupings rather than distinct natural categories. The two-class LCA solution should likewise be regarded as a parsimonious exploratory representation, with the three-class solution remaining a plausible alternative.

One of the most distinctive findings was that the needle-focused and procedural trigger-content profiles did not differ in overall RCT fear or VAS-A distributions. These profiles therefore distinguished the aspects of treatment participants feared rather than separating participants by overall anxiety severity. Most participants in both profiles endorsed only one trigger, so the broader trigger range of the procedural profile should be interpreted as a class-level response pattern rather than as evidence of multiple simultaneous fears in most members. Fear severity patterns and trigger-content profiles thus captured complementary rather than interchangeable dimensions.

Most measured negative RCT experiences and indirect exposures also showed similar distributions across the two trigger-content profiles. The only observed difference was a higher frequency of negative RCT experience in childhood in the procedural profile; however, this comparison was based on a single affirmative response in the needle-focused profile. It should therefore be considered exploratory and may reflect sparse data rather than a reproducible distinction. The overall pattern suggests that the profiles primarily distinguish the content of fear rather than defining stable etiological groups based on previous experiences or exposures.

The high-fear pattern reflected more than elevated VAS-A scores. Participants in this pattern reported more pronounced fear when thinking about visiting the dentist and when sitting in the dental chair, a greater number of RCT triggers, less frequent regular dental attendance, more common postponement behavior, and more negative RCT experiences in childhood and adulthood. In the adjusted model, female sex and negative RCT experience in adulthood were associated with greater odds of high-fear pattern membership, whereas regular dental attendance was associated with lower odds. Negative childhood RCT experience and postponement showed positive but non-significant adjusted associations, and age group was not independently associated with pattern membership. These findings are consistent with the fear-avoidance cycle linking dental fear with service use [8,9] and with adult evidence relating dental anxiety to unpleasant experiences and behavioral reactions [7]. However, the cross-sectional design does not establish direction or causality.

The proportion with severe dental anxiety was approximately four times higher among women than men (35.8% vs. 8.2%), and women were more strongly represented in the high-fear pattern. This finding is consistent with previous studies reporting higher levels of dental fear and anxiety among women [2,5,6,20,36] and with recent observations of anxiety immediately before endodontic treatment [19]. Although participants aged 26–45 years were more strongly represented in the high-fear pattern, the overall age-group comparison was at the conventional significance boundary (*p* = 0.050), and VAS-A classification did not differ across age groups. The age finding should therefore be regarded as hypothesis-generating rather than as a reproducible general age effect.

The proportion with severe dental anxiety increased descriptively from 1.9% among participants reporting no trigger to 54.3% among those reporting three or more triggers. Similarly, this proportion increased from 9.3% among those reporting no source of negative experiences or exposures to 50.0% among those reporting two or more sources. Because these findings were based on omnibus categorical comparisons rather than a formal trend model, they should not be interpreted as evidence of a linear trend or dose–response relationship. They indicate that high anxiety co-occurred with multiple triggers and negative sources within the same participant [9,14,15,16,37], but do not establish the independent contribution of individual triggers or a causal effect of cumulative exposure.

Personal negative experiences and social or media exposures do not represent the same psychological pathway. Personal negative RCT experiences may be considered within a direct conditioning framework; experiences conveyed by family or the social environment through observational learning; and media content through verbal and indirect pathways of fear acquisition [9,12,14,15,16]. More than half of those reporting negative experiences in childhood, adulthood, or the social environment stated that these experiences contributed to their current fear. Although negative media exposure was less frequent (10.5%), 78.3% of the 23 exposed patients stated that such content contributed to their current fear. Because this proportion is based on the retrospective subjective assessment of a small subgroup, it does not demonstrate that the media caused the fear or exerted a stronger influence than other sources.

Previous RCT experience was not associated with endorsement of at least one RCT fear trigger. Descriptively, fear of pain during and after the procedure was reported more often among participants without previous RCT experience, whereas instrument sounds and prolonged mouth opening were reported more often among those with previous RCT experience. These patterns suggest that not only the presence of experience but also the procedural elements with which it is remembered may be important [18,21,22,25,38]. However, previous RCT experience was recorded only as a binary variable, without information on timing, frequency, perceived quality, pain, or treatment outcome. These differences should therefore be interpreted descriptively.

The nearly equal proportions of participants selecting tooth extraction and RCT as the most frightening dental procedure are also noteworthy. Although RCT is intended to preserve the tooth, its being perceived as nearly as frightening as extraction suggests that it remains associated in patients’ minds with pain, threat, procedural duration, and uncertainty. This finding is consistent with previous studies reporting that RCT is perceived as a painful or anxiety-provoking procedure [17,18,25,38,39].

The functions of the measurements used should be distinguished when interpreting the findings. The VAS-A assessed the severity of situational preoperative anxiety, whereas the structured questionnaire assessed fear related to the dental setting, endodontic-specific triggers, sources of negative experiences or exposures, and avoidance behavior. Items concerning thinking about visiting the dentist and sitting in the dental chair were consistent with the anticipatory and clinical setting dimensions in the DAS/MDAS tradition; endodontic-specific triggers were consistent with the DFS, Endodontic Fear Survey, and studies of RCT perceptions [1,18,25,26,27,38]. This structure allowed situational preoperative anxiety severity and fear content to be examined within the same framework but as separate dimensions. Although Facco et al. [29] associated the ≥7.0 cm threshold with phobic dental anxiety, dental phobia requires clinical evaluation of persistence, disproportionality, avoidance, and functional impairment [3,4]. Accordingly, this category was used as a non-diagnostic label of “severe dental anxiety” in the present study. The findings should not be interpreted as evidence of the psychometric validity of the complete questionnaire.

From a practical perspective, a brief two-step chairside assessment could combine a rapid severity measure such as the VAS-A with a short checklist of specific RCT triggers. A predominantly needle-focused response might prompt measures such as reducing visual exposure to the syringe, topical anesthetic use, slow injection, distraction, and explicit confirmation of local anesthetic effectiveness. A broader procedural profile might instead prompt stepwise explanation, anticipatory guidance about sensations and sounds, an agreed stop signal, planned pauses, and reinforcement of the patient’s sense of control. These profile-informed adaptations are clinically plausible but were not tested in the present study [32,40].

The findings should be interpreted in light of several considerations. First, the study was conducted at a single university endodontic clinic within one Turkish healthcare setting. Cultural beliefs about RCT, access and referral pathways, treatment costs, and previous dental care experiences may differ across populations and healthcare systems, which may limit the transferability of the findings. In addition, 178 of 425 invited patients (41.9%) declined participation. Because demographic and clinical information for non-participants was not retained in a form that allowed comparison with the analytical sample, some degree of volunteer or selection bias is possible, and the observed prevalence estimates and the distributions of the identified patterns and profiles may not fully reflect all eligible patients. Similarly, the excluded questionnaires could not be compared with the final analytical sample beyond the recorded data quality reasons. Pulpal and apical diagnoses, acute pain, analgesic use, urgency of treatment, tooth type, and other preoperative clinical characteristics were not predefined study variables; therefore, their potential influence on anxiety and pattern/profile membership could not be examined. Because comparative information was unavailable for non-participants, the direction and magnitude of potential selection bias could not be quantified.

Second, the structured questionnaire was not designed as a scale development or psychometric validation study. It drew on items and concepts from established dental anxiety and endodontic fear instruments and was used as a multidimensional descriptive tool to support fear profiling rather than as a formally validated standalone psychometric scale. Formal pilot testing, construct validation, and test-retest reliability assessment were not performed. Accordingly, the present findings do not establish the psychometric properties of the complete questionnaire. Excluding internally inconsistent questionnaires improved logical coherence of the dataset, but it may also have removed some participants who were uncertain, ambivalent, confused by conditional items, or highly anxious. Previous RCT experience was recorded as present or absent, and variation in its timing, frequency, perceived quality, associated pain, and treatment outcome could therefore not be explored. Because fear triggers were recorded as multiple-response items that were not mutually exclusive, their frequencies describe co-occurring concerns rather than their independent or relative contributions to overall fear. Although the VAS-A categories were prespecified using published thresholds, categorizing the continuous score reduced information and made the clustering partly dependent on the selected cutoffs. Retrospective self-report of previous experiences and their perceived contributions is susceptible to recall and attribution bias; the direction and magnitude of these biases cannot be determined from the present data.

Third, the fear severity patterns and trigger-content profiles should be viewed as exploratory. The revised primary HCA removed the redundant binary trigger-endorsement indicator and used five nonredundant indicators. Although the Gower-based analysis recovered the same broad lower- versus higher-fear structure, differences in cluster sizes and participant assignments indicated sensitivity to the distance measure and clustering algorithm. For LCA, the model-selection indices were not fully concordant, and the three-class solution remained a plausible alternative. The smaller needle-focused profile, boundary item-response estimates, its moderate average posterior probability, and six bivariate residuals above 3.84 indicate model and classification uncertainty. External comparisons based on modal assignment may also have been attenuated or distorted by classification error. In addition, the a priori sample-size calculation was designed for selected chi-square comparisons rather than HCA, LCA, or multivariable regression and does not establish the adequacy or stability of these exploratory models. Replication and external validation in larger, independent, and culturally diverse samples are needed. Finally, the multivariable analysis remains observational and cross-sectional; its associations do not establish temporal or causal relationships. These adjusted estimates should not be interpreted as mediated effects or causal pathways.

Overall, the findings suggest that the severity of RCT fear, its dominant triggers, sources of negative experiences or exposures, and dental avoidance behavior are related but non-interchangeable dimensions. The person-centered approach indicates that participants with similar anxiety levels may report different fear contents.

Future multicenter and prospective studies in independent and culturally diverse samples should evaluate the reproducibility and stability of the fear severity patterns and trigger-content profiles and clarify the temporal relationships among clinical status, negative experiences, fear severity, trigger content, and avoidance behavior. Future studies should also test whether brief trigger-focused screening and profile-informed anxiety management improve patient experience, treatment attendance, pain-related outcomes, or clinical care. Longitudinal designs with repeated, temporally ordered measurements and a prespecified causal framework would be required to evaluate mediation or pathway hypotheses.

## 5. Conclusions

Among adult endodontic patients, fear of RCT showed exploratory fear severity patterns and trigger-content profiles. Severity-based analysis produced low- and high-fear patterns, whereas trigger-content analysis yielded needle-focused and procedural profiles. These classifications describe complementary dimensions of the present sample and should not be interpreted as fixed clinical categories.

Participants with similar anxiety levels could fear different aspects of treatment, indicating that total anxiety severity alone did not fully characterize fear content. In some participants, fear was concentrated on injections and needles; in others, anticipated pain, prolonged mouth opening, instrument sensations and sounds, postoperative symptoms, and the possibility of treatment failure occurred across a broader trigger pattern. The proportion with severe dental anxiety increased descriptively across higher trigger-count and negative-source categories, but no formal trend analysis was performed, and causal or trigger-specific contributions cannot be inferred.

In the adjusted analysis, female sex and negative RCT experience in adulthood were associated with higher odds of high-fear pattern membership, whereas regular dental attendance was associated with lower odds. Negative childhood RCT experience and postponement of dental visits were not independently significant. A combined assessment of anxiety severity, specific triggers, previous experiences, and attendance behavior may help clinicians individualize communication and supportive care. The trigger-content profiles require external validation, and whether profile-informed interventions improve clinical outcomes requires prospective evaluation.

## Figures and Tables

**Table 1:** Hierarchical multivariable logistic regression analysis of factors associated with high-fear pattern membership.

Predictor/Contrast	Model 1		Model 2		Model 3	
	AOR (95% CI)	*p* (Z)	AOR (95% CI)	*p* (Z)	AOR (95% CI)	*p* (Z)
**Sex**						
Male *(ref)*						
Female	3.598 (1.907–6.787)	*<0.001* (3.954)	3.962 (2.038–7.703)	*<0.001* (4.059)	4.265 (2.138–8.506)	*<0.001* (4.118)
**Age group**						
18–25 years *(ref)*						
26–35	1.491 (0.673–3.301)	0.325 (0.984)	1.483 (0.652–3.369)	0.347 (0.940)	1.269 (0.534–3.015)	0.589 (0.540)
36–45	1.186 (0.492–2.857)	0.704 (0.380)	1.542 (0.611–3.888)	0.359 (0.917)	1.296 (0.479–3.505)	0.609 (0.511)
46–55	0.443 (0.140–1.402)	0.166 (−1.385)	0.472 (0.142–1.571)	0.221 (−1.223)	0.477 (0.139–1.640)	0.240 (−1.174)
56–60	0.447 (0.129–1.553)	0.205 (−1.267)	0.525 (0.144–1.910)	0.328 (−0.978)	0.560 (0.151–2.069)	0.384 (−0.870)
**Regular dental attendance**						
No *(ref)*						
Yes	-	-	0.409 (0.194–0.863)	*0.019* (−2.347)	0.393 (0.182–0.846)	*0.017* (−2.386)
**Postponement of dental visits**						
No *(ref)*						
Yes	-	-	2.087 (0.969–4.495)	0.060 (1.879)	2.083 (0.943–4.599)	0.070 (1.815)
Cannot recall	-	-	1.570 (0.119–20.641)	0.732 (0.343)	1.601 (0.119–21.622)	0.723 (0.354)
**Negative RCT experience in childhood**						
No *(ref)*						
Yes	-	-	-	-	2.517 (0.942–6.724)	0.066 (1.842)
Cannot recall	-	-	-	-	0.593 (0.095–3.692)	0.576 (−0.560)
**Negative RCT experience in adulthood**						
No *(ref)*						
Yes	-	-	-	-	2.187 (1.002–4.773)	*0.049* (1.966)
Cannot recall *	-	-	-	-	Not estimable	—

Note. Hierarchical binary logistic regression; N = 219 (high-fear n = 67; low-fear n = 152); outcome coding: high-fear = 1 and low-fear = 0. Model 1: sex and age group. Model 2: Model 1 plus regular dental attendance and postponement. Model 3: Model 2 plus negative RCT experiences in childhood and adulthood. Reference categories are marked ref. AOR, adjusted odds ratio; CI, confidence interval; RCT, root canal treatment. * Adult ‘cannot recall’ contained one participant; its category-specific AOR, 95% CI, and *p*-value were not estimable or interpreted. Remaining maximum-likelihood estimates are shown as fitted. Italic type is used for statistical symbols (*p* and Z), reference-category labels (ref), and statistically significant *p*-values (*p* < 0.05).

**Table 2:** Comparison of the latent class models and item-response probabilities in the retained two-class solution.

**(A) Model Fit Indices**						
**Model**	**Log-** **Likelihood**	**Free** **Parameters**	**AIC**	**BIC**	**Entropy**	**Estimated Class Proportions**
2-class solution (retained)	−626.81	17	1287.62	1340.53	0.833	17.7%/82.3%
3-class solution	−610.77	26	1273.54	1354.45	0.948	64.5%/12.4%/23.2%
**(B) Item-Response Probabilities in the Retained Two-Class Solution**						
**RCT Fear Trigger**			**Needle-Focused Profile**		**Procedural Profile**	
Injection/needle fear			1.000		0.217	
Fear of pain during treatment			0.147		0.510	
Prolonged mouth opening			0.000		0.344	
Fear of postoperative pain			0.046		0.210	
Possibility of treatment failure			0.000		0.190	
Sensation of endodontic instruments			0.000		0.161	
Instrument sounds			0.000		0.146	
Fear of postoperative swelling			0.000		0.095	

Note: AIC, Akaike information criterion; BIC, Bayesian information criterion; RCT, root canal treatment. Lower AIC and BIC values indicate better relative fit, whereas higher entropy indicates clearer class separation; entropy was not used as the sole selection criterion. Estimated class proportions are model-based. Maximum posterior modal assignment in the retained solution yielded n = 38 (22.9%) and n = 128 (77.1%). Panel B reports conditional item-response probabilities. Values of 0.000 and 1.000 are boundary estimates and should be interpreted cautiously. The two-class solution was retained based on lower BIC, class size, parsimony, and clinical interpretability.

**Table 3:** Clinical expression and negative RCT experiences or exposures according to modally assigned trigger-content profile.

Characteristic/Category	Needle-Focused Profile (n = 38)	Procedural Profile (n = 128)	*p*-Value
**A. Clinical expression of RCT fear**			
Fear at the prospect of undergoing RCT			
None	2 (5.3)	10 (7.8)	0.915
Low	9 (23.7)	34 (26.6)	
Moderate	18 (47.4)	52 (40.6)	
High	7 (18.4)	22 (17.2)	
Very high	2 (5.3)	10 (7.8)	
VAS-A classification			
No/low dental anxiety	18 (47.4)	60 (46.9)	0.693
Dental anxiety	11 (28.9)	30 (23.4)	
Severe dental anxiety	9 (23.7)	38 (29.7)	
**B. Negative RCT experiences**			
Negative RCT experience in childhood			
Yes	1 (2.6)	22 (17.2)	0.042
Cannot recall	2 (5.3)	5 (3.9)	
No	35 (92.1)	101 (78.9)	
Negative RCT experience in adulthood			
Yes	7 (18.4)	43 (33.6)	0.128
Cannot recall	0 (0.0)	1 (0.8)	
No	31 (81.6)	84 (65.6)	
**C. Indirect experiences and exposures**			
Negative RCT experience among family members or within the social environment			
Yes	8 (21.1)	36 (28.1)	0.379
Cannot recall	7 (18.4)	31 (24.2)	
No	23 (60.5)	61 (47.7)	
Negative media exposure related to RCT			
Yes	4 (10.5)	16 (12.5)	0.245
Cannot recall	4 (10.5)	28 (21.9)	
No	30 (78.9)	84 (65.6)	

Note: Values are presented as n (%) using column percentages. Class membership for these exploratory external comparisons was based on maximum posterior (modal) assignment. *p*-values refer to the complete category distributions shown in the table. Pearson’s chi-square or Fisher’s exact test was used as appropriate. The childhood experience comparison should be interpreted cautiously because only one participant in the needle-focused profile reported a negative childhood RCT experience. RCT, root canal treatment; VAS-A, Visual Analog Scale for Anxiety.

## Data Availability

The data presented in this study are available from the corresponding author upon reasonable request because of confidentiality and ethical restrictions. The data are not publicly available because they include participant-level questionnaire information.

## Supplementary Materials

The following supporting information can be downloaded at https://www.mdpi.com/article/10.3390/healthcare14152360/s1: File S1. Questionnaire and Coding Details. File S2. Additional Methodological and Statistical Details. File S3. Completed STROBE Checklist for Cross-Sectional Studies.

## Abbreviations

The following abbreviations are used in this manuscript:

AIC	Akaike information criterion
BIC	Bayesian information criterion
CI	Confidence interval
DAS	Dental Anxiety Scale
DFS	Dental Fear Survey
HCA	Hierarchical cluster analysis
LCA	Latent class analysis
MDAS	Modified Dental Anxiety Scale
RCT	Root canal treatment
VAS-A	Visual Analog Scale for Anxiety

## References

[1] Corah N.L. (1969). Development of a Dental Anxiety Scale. J. Dent. Res..

[2] Armfield J., Spencer A., Stewart J. (2006). Dental Fear in Australia: Who’s Afraid of the Dentist?. Aust. Dent. J..

[3] Oosterink F.M.D., De Jongh A., Hoogstraten J. (2009). Prevalence of Dental Fear and Phobia Relative to Other Fear and Phobia Subtypes. Eur. J. Oral Sci..

[4] Seligman L.D., Hovey J.D., Chacon K., Ollendick T.H. (2017). Dental Anxiety: An Understudied Problem in Youth. Clin. Psychol. Rev..

[5] Silveira E.R., Cademartori M.G., Schuch H.S., Armfield J.A., Demarco F.F. (2021). Estimated Prevalence of Dental Fear in Adults: A Systematic Review and Meta-Analysis. J. Dent..

[6] Zinke A., Hannig C., Berth H. (2019). Psychological Distress and Anxiety Compared amongst Dental Patients-Results of a Cross-Sectional Study in 1549 Adults. BMC Oral Health.

[7] Peric R., Tadin A. (2024). Associations Between Dental Anxiety Levels, Self-Reported Oral Health, Previous Unpleasant Dental Experiences, and Behavioural Reactions in Dental Settings: An Adult E-Survey. Medicina.

[8] Armfield J.M., Stewart J.F., Spencer A.J. (2007). The Vicious Cycle of Dental Fear: Exploring the Interplay Between Oral Health, Service Utilization and Dental Fear. BMC Oral Health.

[9] Beaton L., Freeman R., Humphris G. (2014). Why Are People Afraid of the Dentist? Observations and Explanations. Med. Princ. Pract..

[10] Ng S.K.S., Leung W.K. (2008). A Community Study on the Relationship of Dental Anxiety with Oral Health Status and Oral Health-related Quality of Life. Community Dent. Oral Epidemiol..

[11] Carlsson V., Hakeberg M., Wide Boman U. (2015). Associations between Dental Anxiety, Sense of Coherence, Oral Health-Related Quality of Life and Health Behaviour—A National Swedish Cross-Sectional Survey. BMC Oral Health.

[12] Carter A.E., Carter G., Boschen M., AlShwaimi E., George R. (2015). Ethnicity and Pathways of Fear in Endodontics. J. Endod..

[13] Armfield J.M., Slade G.D., Spencer A.J. (2008). Cognitive Vulnerability and Dental Fear. BMC Oral Health.

[14] Rimm D.C., Janda L.H., Lancaster D.W., Nahl M., Dittmar K. (1977). An Exploratory Investigation of the Origin and Maintenance of Phobias. Behav. Res. Ther..

[15] Davey G.C.L. (1989). Dental Phobias and Anxieties: Evidence for Conditioning Processes in the Acquisition and Modulation of a Learned Fear. Behav. Res. Ther..

[16] Field A.P., Ball J.E., Kawycz N.J., Moore H. (2007). Parent-Child Relationships and the Verbal Information Pathway to Fear in Children: Two Preliminary Experiments. Behav. Cogn. Psychother..

[17] Wong M., Lytle W.R. (1991). A Comparison of Anxiety Levels Associated with Root Canal Therapy and Oral Surgery Treatment. J. Endod..

[18] Khan S., Hamedy R., Lei Y., Ogawa R.S., White S.N. (2016). Anxiety Related to Nonsurgical Root Canal Treatment: A Systematic Review. J. Endod..

[19] Busatto P.F., Baldissarelli F., Pelepenko L.E., Vanni J.R., Fornari V.J., Rigo L., Hartmann M.S.M. (2024). Anxiety Immediately Before Endodontic Treatment: Cross-Sectional Quantitative Analysis. Discov. Psychol..

[20] Alghofaily M., Alsalleeh F. (2022). Levels of Anxiety and Fear Related to Non-Surgical Root Canal Treatment Performed by Endodontic Residents and Endodontists. Front. Dent. Med..

[21] Watkins C.A., Logan H.L., Kirchner H.L. (2002). Anticipated and Experienced Pain Associated with Endodontic Therapy. J. Am. Dent. Assoc..

[22] Van Wijk A.J., Hoogstraten J. (2006). Reducing Fear of Pain Associated with Endodontic Therapy. Int. Endod. J..

[23] Dou L., Vanschaayk M.M., Zhang Y., Fu X., Ji P., Yang D. (2018). The Prevalence of Dental Anxiety and Its Association with Pain and Other Variables Among Adult Patients with Irreversible Pulpitis. BMC Oral Health.

[24] Murillo-Benítez M., Martín-González J., Jiménez-Sánchez M.C., Cabanillas-Balsera D., Velasco-Ortega E., Segura-Egea J.J. (2020). Association Between Dental Anxiety and Intraoperative Pain During Root Canal Treatment: A Cross-Sectional Study. Int. Endod. J..

[25] LeClaire A.J., Skidmore A.E., Griffin J.A., Balaban F.S. (1988). Endodontic Fear Survey. J. Endod..

[26] Kleinknecht R.A., Klepac R.K., Alexander L.D. (1973). Origins and Characteristics of Fear of Dentistry. J. Am. Dent. Assoc..

[27] İlgüy D., İlgüy M., Dinçer S., Bayirli G. (2005). Reliability and Validity of the Modified Dental Anxiety Scale in Turkish Patients. J. Int. Med. Res..

[28] Luyk N.H., Beck F.M., Weaver J.M. (1988). A Visual Analogue Scale in the Assessment of Dental Anxiety. Anesth. Prog..

[29] Facco E., Zanette G., Favero L., Bacci C., Sivolella S., Cavallin F., Manani G. (2011). Toward the Validation of Visual Analogue Scale for Anxiety. Anesth. Prog..

[30] Appukuttan D., Vinayagavel M., Tadepalli A. (2014). Utility and Validity of a Single-Item Visual Analog Scale for Measuring Dental Anxiety in Clinical Practice. J. Oral Sci..

[31] Peretz B., Moshonov J. (1998). Dental Anxiety Among Patients Undergoing Endodontic Treatment. J. Endod..

[32] Craveiro M.A., Caldeira C.L. (2020). Influence of an Audiovisual Resource on the Preoperative Anxiety of Adult Endodontic Patients: A Randomized Controlled Clinical Trial. J. Endod..

[33] Kajita M., Pohjola V., Kataja E.-L., Lahti S. (2025). Exploring High Dental Anxiety Subtypes Using Cluster Analysis Approach: A Cross-Sectional Study. BMC Oral Health.

[34] Von Elm E., Altman D.G., Egger M., Pocock S.J., Gøtzsche P.C., Vandenbroucke J.P. (2008). The Strengthening the Reporting of Observational Studies in Epidemiology (STROBE) Statement: Guidelines for Reporting Observational Studies. J. Clin. Epidemiol..

[35] Ward J.H. (1963). Hierarchical Grouping to Optimize an Objective Function. J. Am. Stat. Assoc..

[36] Saba Z., Katirci G. (2023). Relationship Between Dental Anxiety Levels and Oral Health Among Dental Patients in Turkey: A Cross-Sectional Study. BMC Oral Health.

[37] Rachman S., Lopatka C. (1986). Do Fears Summate?—III. Behav. Res. Ther..

[38] Chandraweera L., Goh K., Lai-Tong J., Newby J., Abbott P. (2019). A Survey of Patients’ Perceptions About, and Their Experiences of, Root Canal Treatment. Aust. Endod. J..

[39] Stabholz A., Peretz B. (1999). Dental Anxiety among Patients Prior to Different Dental Treatments. Int. Dent. J..

[40] Silva I.A., Dall Agnol Júnior C.A., Weissheimer T., Reis So M.V., Da Rosa R.A. (2023). Pharmacological Management of Anxiety on Pain Occurrence During Root Canal Treatment: A Systematic Review. Eur. Endod. J..

