# Supplementary material for: Profiles of Fear of Root Canal Treatment in Adult Endodontic Patients: A Cross-Sectional Study
Source: Healthcare (Basel). 2026 Aug 3;14(15):2360. doi: 10.3390/healthcare14152360 (PMC13465031; doi:10.3390/healthcare14152360)
Supplement: Supplementary file 1 [file healthcare-14-02360-s001.zip › Supplementary File S1. Questionnaire and Coding Details.pdf]

Supplementary File S1. Structured Questionnaire, Coding Rules

Note. The questionnaire was administered in Turkish. The English translation is provided for transparency and replication. The 0–10 item is the Visual Analog Scale for Anxiety (VAS-A). The questionnaire pages reproduce the administered form; administration, coding, skip logic, and data-quality definitions follow in Appendices S1A–S1C.

English Translation

1. Gender

☐ Female

☐ Male

2. Age

☐ 18-25

☐ 26-35

☐ 36-45

☐ 46-55

☐ 56-60

3. What is your educational level?

☐ Primary school

☐ Middle school

☐ High school

☐ Undergraduate degree

☐ Postgraduate degree

☐ Other: ______________

4. Do you visit the dentist regularly?

☐ Yes

☐ No

5. Have you ever avoided or postponed visiting the dentist even though you knew that you needed to go?

(If your answer is yes, please answer the next question.)

☐ Yes

☐ No

☐ I do not remember

6. What was your reason for avoiding or postponing the visit?

(More than one option may be selected.)

☐ Fear/anxiety

☐ Cost of dental treatment

☐ Lack of time for dental treatment

☐ No pain complaint/pain not severe enough to be intolerable

☐ Previous negative treatment experience

☐ Negative influence of people around me/the media

7. Which of the following dental treatments causes you the most fear?

☐ Tooth extraction

☐ Root canal treatment

☐ Filling

☐ Prosthetic treatment

☐ Dental cleaning

☐ Dental examination

☐ Orthodontic treatment

8. How much fear does the thought of visiting the dentist cause you?

☐ None

☐ A little

☐ Moderate

☐ A lot

☐ Very much

9. How much fear does sitting in the dental chair cause you?

☐ None

☐ A little

☐ Moderate

☐ A lot

☐ Very much

10. Have you previously undergone root canal treatment?

☐ Yes

☐ No

11. Which of the following causes you fear during root canal treatment?

(More than one option may be selected.)

☐ I have no fear

☐ Injection/needle

☐ Fear of feeling pain during treatment

☐ Fear of feeling pain after treatment

☐ Fear of swelling after treatment

☐ Sounds produced by the instruments during treatment

☐ Sensation caused by root canal instruments

☐ Having to keep the mouth open for a long time

☐ Possibility of treatment failure

☐ Other: __________

12. Did you have a negative root canal treatment experience during childhood?

(If your answer is yes, please answer the next question.)

☐ Yes

☐ No

☐ I do not remember

13. Do you think that your negative childhood experience contributes to your current fear of root canal treatment?

☐ Yes

☐ No

☐ I am not sure

14. Have you had a negative root canal treatment experience during adulthood?

(If your answer is yes, please answer the next question.)

☐ Yes

☐ No

☐ I do not remember

15. Do you think that your negative experience during adulthood contributes to your current fear of root canal treatment?

☐ Yes

☐ No

☐ I am not sure

16. Have any members of your family, relatives, or people around you had a negative root canal treatment experience?

(If your answer is yes, please answer the next question.)

☐ Yes

☐ No

☐ I do not remember

17. Do you think that this contributes to your current fear of root canal treatment?

☐ Yes

☐ No

☐ I am not sure

18. Have you encountered negative information about root canal treatment in the media?

(If your answer is yes, please answer the next question.)

☐ Yes

☐ No

☐ I do not remember

19. Do you think that this contributes to your current fear of root canal treatment?

☐ Yes

☐ No

☐ I am not sure

20. Regardless of any negative experiences or information you have heard, how much fear does the thought of undergoing root canal treatment cause you?

☐ None

☐ A little

☐ Moderate

☐ A lot

☐ Very much

21. What is your level of fear before the procedure?

0 ____ 10

Original Turkish Version

1. Cinsiyet

☐ Kadın

☐ Erkek

2. Yaş

☐ 18-25

☐ 26-35

☐ 36-45

☐ 46-55

☐ 56-60

3. Eğitim durumunuz nedir?

☐ İlkokul

☐ Ortaokul

☐ Lise

☐ Lisans

☐ Lisansüstü

☐ Diğer: _______________

4. Düzenli olarak diş hekimine gider misiniz?

☐ Evet

☐ Hayır

5. Diş hekimine gitmeniz gerektiğini bildiğiniz hâlde gitmediğiniz/ertelediğiniz oldu mu?

(Cevabın evet olması hâlinde bir sonraki soru cevaplanacaktır.)

☐ Evet

☐ Hayır

☐ Hatırlamıyorum

6. Gitmeme/erteleme sebebiniz neydi?

(Birden fazla seçenek işaretlenebilmektedir.)

☐ Korku/anksiyete

☐ Diş tedavisinin maliyeti

☐ Diş tedavisine ayıracak zamanın bulunmaması

☐ Ağrı şikayetinin bulunmaması/ağrı şikayetinin dayanılamayacak bir şiddette olmaması

☐ Geçmişteki olumsuz tedavi tecrübesi

☐ Çevrenin/medyanın olumsuz etkisi

7. Aşağıdaki diş tedavilerinden hangisi sizde daha fazla korkuya sebep olur?

☐ Diş çekimi

☐ Kanal tedavisi

☐ Dolgu

☐ Protez

☐ Diş temizliği

☐ Muayene

☐ Tel tedavisi

8. Diş hekimine gideceğinizi düşünmek sizde ne kadar korkuya sebep olur?

☐ Hiç

☐ Az

☐ Orta

☐ Fazla

☐ Çok fazla

9. Diş hekimi koltuğuna oturmak sizde ne kadar korkuya sebep olur?

☐ Hiç

☐ Az

☐ Orta

☐ Fazla

☐ Çok fazla

10. Daha önce kanal tedavisi yaptırdınız mı?

☐ Evet

☐ Hayır

11. Kök kanal tedavisi esnasında hangisi/hangileri sizde korkuya sebep olur?

(Birden fazla seçenek işaretlenebilmektedir.)

☐ Korkum yok

☐ Enjeksiyon/İğne

☐ Tedavi esnasında ağrı hissetme korkusu

☐ Tedavi sonrasında ağrı hissetme korkusu

☐ Tedavi sonrasında şişlik olması korkusu

☐ Tedavi esnasında aletlerin çıkardıkları sesler

☐ Kanal aletlerinin vermiş olduğu his

☐ Uzun süre ağzı açık tutmanın gerekmesi

☐ Tedavinin başarısız olma ihtimali

☐ Diğer: __________

12. Çocukluğunuzda olumsuz bir kanal tedavisi tecrübeniz oldu mu?

(Cevabın evet olması hâlinde bir sonraki soru cevaplanacaktır.)

☐ Evet

☐ Hayır

☐ Hatırlamıyorum

13. Çocukluğunuzdaki olumsuz tecrübenin bugün kanal tedavisi korkusuna sebep olduğunu düşünüyor musunuz?

☐ Evet

☐ Hayır

☐ Emin değilim

14. Yetişkinliğinizde olumsuz bir kanal tedavisi tecrübeniz oldu mu?

(Cevabın evet olması hâlinde bir sonraki soru cevaplanacaktır.)

☐ Evet

☐ Hayır

☐ Hatırlamıyorum

15. Yetişkinliğinizdeki olumsuz tecrübenin bugün kanal tedavisi korkusuna sebep olduğunu düşünüyor musunuz?

☐ Evet

☐ Hayır

☐ Emin değilim

16. Aile, akraba ya da çevrenizdeki kişilerin olumsuz bir kanal tedavisi tecrübesi oldu mu?

(Cevabın evet olması hâlinde bir sonraki soru cevaplanacaktır.)

☐ Evet

☐ Hayır

☐ Hatırlamıyorum

17. Bu durumun bugün kanal tedavisi korkusuna sebep olduğunu düşünüyor musunuz?

☐ Evet

☐ Hayır

☐ Emin değilim

18. Medya kanallarında kanal tedavisiyle alakalı olumsuz bilgilerle karşılaştınız mı?

(Cevabın evet olması hâlinde bir sonraki soru cevaplanacaktır.)

☐ Evet

☐ Hayır

☐ Hatırlamıyorum

19. Bu durumun bugün kanal tedavisi korkusuna sebep olduğunu düşünüyor musunuz?

☐ Evet

☐ Hayır

☐ Emin değilim

20. Tüm olumsuz tecrübeler ve duyduklarınızdan bağımsız olarak size kanal tedavisi yapılacağını düşünmek sizde ne kadar korkuya sebep olur?

☐ Hiç

☐ Az

☐ Orta

☐ Fazla

☐ Çok fazla

21. İşlem öncesindeki korku seviyeniz nedir?

0 ____ 10

Section S1A. Administration and instrument status

The Turkish questionnaire was completed before primary root canal treatment. A study investigator provided the form, and participants completed it independently without guidance from the clinician who would perform the procedure. Only technical instructions on how to mark a response were given; no explanation intended to influence response content was provided.

The English version is a reporting translation and was not separately administered. The questionnaire was used as a multidimensional descriptive tool; it was not treated as a validated standalone psychometric scale.

No preliminary cognitive interviews, informal pilot administration, or formal pilot testing was performed before data collection. Construct validation and test–retest reliability assessment were also not performed.

Table S1. Item-level response options and analytical coding

Item/domain	Response options	Coding and use
Q1. Sex	Female; male	Categorical. In regression, female was the reference and the fitted contrast was male vs. female.
Q2. Age	18–25; 26–35; 36–45; 46–55; 56–60 years	Categorical; 18–25 years was the regression reference.

Q3. Education	Primary; middle; high school; undergraduate; postgraduate; other	Categorical descriptive variable.
Q4. Regular dental attendance	Yes; no	Binary categorical variable; no was the regression reference.
Q5. Avoided/postponed a needed dental visit	Yes; no; do not remember	Categorical. Regression contrasts: yes and cannot recall vs. no.
Q6. Reason for avoidance/postponement	Multiple responses permitted	Descriptive multiple-response item; completed only when Q5 = Yes.
Q7. Most frightening dental treatment	Extraction; RCT; filling; prosthetic; cleaning; examination; orthodontic	Single-choice descriptive item.
Q8. Fear when thinking about visiting the dentist	None; a little; moderate; a lot; very much	Ordinal 0–4 in the same order; HCA indicator.
Q9. Fear when sitting in the dental chair	None; a little; moderate; a lot; very much	Ordinal 0–4 in the same order; HCA indicator.
Q10. Previous RCT	Yes; no	Binary categorical variable; no was the regression reference.
Q11. RCT fear triggers	No fear; eight prespecified triggers; Other (free text)	Multiple response. Each of the eight trigger indicators used in the analysis was coded 1 if endorsed and 0 otherwise. See Section S1B.
Q12. Negative RCT experience in childhood	Yes; no; do not remember	Categorical external characteristic; no was the regression reference.
Q13. Childhood experience contributes to current fear	Yes; no; not sure	Conditional descriptive attribution item; answered only when Q12 = Yes.
Q14. Negative RCT experience in adulthood	Yes; no; do not remember	Categorical external characteristic; no was the regression reference.
Q15. Adulthood experience contributes to current fear	Yes; no; not sure	Conditional descriptive attribution item; answered only when Q14 = Yes.
Q16. Negative RCT experience in family/social environment	Yes; no; do not remember	Categorical source/exposure indicator.
Q17. Family/social experience contributes to current fear	Yes; no; not sure	Conditional descriptive attribution item; answered only when Q16 = Yes.
Q18. Negative media information about	Yes; no; do not remember	Categorical media-exposure indicator.

RCT		
Q19. Media exposure contributes to current fear	Yes; no; not sure	Conditional descriptive attribution item; answered only when Q18 = Yes.
Q20. Fear at the prospect of undergoing RCT	None; a little; moderate; a lot; very much	Ordinal 0–4 in the same order; HCA indicator.
Q21. Preoperative anxiety (VAS-A)	0–10 cm visual analog line	Continuous distance in cm. Categories: 0–5.0 = no/low dental anxiety; 5.1–6.9 = dental anxiety; ≥7.0 = severe dental anxiety. Category used in HCA.

Note. RCT, root canal treatment; HCA, hierarchical cluster analysis; VAS-A, Visual Analog Scale for Anxiety.

Table S2. Conditional skip logic

Conditional pair	Rule
Q5 → Q6	Complete Q6 only when Q5 = Yes; otherwise proceed to Q7.
Q12 → Q13	Complete Q13 only when Q12 = Yes; otherwise proceed to Q14.
Q14 → Q15	Complete Q15 only when Q14 = Yes; otherwise proceed to Q16.
Q16 → Q17	Complete Q17 only when Q16 = Yes; otherwise proceed to Q18.
Q18 → Q19	Complete Q19 only when Q18 = Yes; otherwise proceed to Q20.

Section S1B. Trigger derivations and person-centered analyses

Derived variable	Definition	Analytical use
Eight dichotomous trigger indicators	Injection/needle; pain during treatment; postoperative pain; postoperative swelling; instrument sounds; sensation of endodontic instruments; prolonged mouth opening; possibility of treatment failure.	Each coded 1 if endorsed and 0 if not endorsed; these eight indicators entered the LCA.
Any endorsed RCT fear trigger	At least one coded RCT fear trigger.	Binary descriptive variable. Final fear-positive sample n = 166.
Observed trigger count	Number of endorsed RCT fear triggers.	Natural count for descriptive tables; grouped as 0, 1, 2, or ≥3 for HCA.
HCA nonredundant indicator set	Q8, Q9, grouped trigger count, Q20, and VAS-A category.	Five indicators z-standardized before Ward–Euclidean HCA. The binary any-trigger variable was excluded.
LCA indicator set	Eight prespecified Q11 triggers among participants with any endorsed trigger.	Two- and three-class exploratory LCA models; n = 166.

Negative-source count	Affirmative responses to Q12, Q14, Q16, and Q18.	Sum 0–4. 'No' and 'do not remember' were not counted as affirmative sources.

Note. The HCA trigger-count variable and the eight dichotomous LCA indicators were derived from the finalized trigger coding used for the revised analyses.

Section S1C. Data-quality definitions and recorded exclusions

**Category/rule**	**Operational definition**	**Recorded number**
Incomplete questionnaire	One or more responses required for the principal analyses were missing, including a required conditional follow-up after an affirmative parent item.	6 unique questionnaires
Previous-RCT contradiction	Q10 indicated no previous RCT while a childhood or adulthood item reported a previous negative RCT experience, or its conditional attribution item was completed affirmatively.	Rule-specific overlapping count not retained
Conditional experience/exposure contradiction	A parent item (Q12, Q14, Q16, or Q18) was marked No/Do not remember, but any response option was marked on its conditional attribution item (Q13, Q15, Q17, or Q19), contrary to the skip instruction.	Rule-specific overlapping count not retained
At least one internal inconsistency	A questionnaire met one or both internal-consistency rule families above. A questionnaire could meet more than one rule.	22 unique questionnaires
No fear plus prespecified trigger	Checked as a verification condition because 'I have no fear' was intended to be mutually exclusive with a trigger. This condition was not used as an exclusion rule.	0 questionnaires in the final cleaned dataset

Total excluded	Incomplete or internally inconsistent questionnaire, counted once at questionnaire level.	28 unique questionnaires

Note. The exclusion log retained unique questionnaire-level outcomes (incomplete, internally inconsistent, or retained) rather than separate binary flags for every internal-consistency rule. Rule-specific overlapping counts therefore cannot be reconstructed reliably from the final anonymized analytical file and are not estimated retrospectively. Exclusion of internally inconsistent forms may have selected against participants who were uncertain, ambivalent, confused by conditional items, or highly anxious.
