# Supplementary material for: Profiles of Fear of Root Canal Treatment in Adult Endodontic Patients: A Cross-Sectional Study
Source: Healthcare (Basel). 2026 Aug 3;14(15):2360. doi: 10.3390/healthcare14152360 (PMC13465031; doi:10.3390/healthcare14152360)
Supplement: Supplementary file 1 [file healthcare-14-02360-s001.zip › Supplementary File S2. Additional Methodological and Statistical Details.pdf]

Supplementary File S2. Additional Methodological and Statistical Details

Table S3. Nonredundant hierarchical cluster analysis indicators and coding before standardization

Indicator	Type	Coding/categories	Direction
Fear when thinking about visiting the dentist	Ordinal	1 = none; 2 = a little; 3 = moderate; 4 = a lot; 5 = very much	Higher = greater fear
Fear when sitting in the dental chair	Ordinal	1 = none; 2 = a little; 3 = moderate; 4 = a lot; 5 = very much	Higher = greater fear
Number of endorsed RCT fear triggers	Grouped count	0 = none; 1 = one; 2 = two; 3 = three or more	Higher = broader endorsement
Fear at the prospect of undergoing RCT	Ordinal	1 = none; 2 = a little; 3 = moderate; 4 = a lot; 5 = very much	Higher = greater fear
VAS-A classification	Ordinal	1 = no/low; 2 = dental anxiety; 3 = severe dental anxiety	Higher = greater anxiety

Note. All five indicators were coded in the same direction and z-standardized before Ward–Euclidean clustering. The binary indicator 'endorsement of at least one RCT fear trigger' was excluded because it was deterministically derived from the trigger-count variable. VAS-A was retained in its prespecified three-category form; the information loss from categorization is acknowledged in the manuscript.

Table S4. Hierarchical cluster analysis internal validation, mixed-data robustness, and participant-level agreement

Analysis	Indicators	Cluster sizes	Average silhouette
Ward–Euclidean, two clusters (primary)	Five nonredundant indicators	152 / 67	0.445
Ward–Euclidean, three clusters	Five nonredundant indicators	124 / 28 / 67	0.390
Gower–average, two clusters (robustness)	Five nonredundant indicators	172 / 47	0.569

Note. In the Ward hierarchy, the n = 124 and n = 28 branches merge to form the retained n = 152 lower-fear pattern; the n = 67 branch corresponds to the retained higher-fear pattern. The two-cluster solution had the higher silhouette coefficient after removal of the redundant indicator.

**Participant-level agreement measure**	**Value**
Adjusted Rand index	0.619
Overall Jaccard coefficient	0.744
Cluster-specific Jaccard: lower-fear correspondence	0.873
Cluster-specific Jaccard: higher-fear correspondence	0.676
Corresponding participant assignments	197/219 (90.0%)

**Ward–Euclidean assignment**	**Gower lower-fear**	**Gower higher-fear**	**Total**
Ward lower-fear pattern (n = 152)	151	1	152
Ward higher-fear pattern (n = 67)	21	46	67
**Column total**	**172**	**47**	**219**

Note. The broad lower- versus higher-fear structure was retained, but the cross-tabulation and agreement coefficients show that exact membership was partly sensitive to the distance measure and clustering algorithm.

Table S5. Latent class model-fit indices and convergence settings

Model	Log-likelihood	Parameters	AIC	BIC	Entropy	Estimated proportions
Two-class solution (retained)	−626.81	17	1287.62	1340.53	0.833	17.7% / 82.3%
Three-class solution	−610.77	26	1273.54	1354.45	0.948	64.5% / 12.4% / 23.2%

Note. Models were estimated among n = 166 participants using 100 random starting values, a maximum of 5000 iterations, and a convergence tolerance of 1 × 10−8. The retained two-class model was re-estimated independently five times with 100 random starts per run; every run reproduced the maximum log-likelihood of −626.81. BIC was the primary parsimony criterion; AIC, entropy, class size, and clinical coherence were considered jointly.

Table S6. Retained two-class LCA: item-response probabilities and observed trigger counts

Prespecified RCT fear trigger	Needle-focused profile	Procedural profile
Injection/needle fear	1.000	0.217
Fear of pain during treatment	0.147	0.510
Prolonged mouth opening	0.000	0.344
Fear of postoperative pain	0.046	0.210
Possibility of treatment failure	0.000	0.190
Sensation of endodontic instruments	0.000	0.161
Instrument sounds	0.000	0.146
Fear of postoperative swelling	0.000	0.095

Observed number of triggers	Needle-focused, n (%)	Procedural, n (%)
1	28 (73.7)	70 (54.7)
2	10 (26.3)	23 (18.0)
3	0 (0.0)	20 (15.6)
4	0 (0.0)	10 (7.8)
5	0 (0.0)	3 (2.3)
6	0 (0.0)	2 (1.6)

Note. Modal assignment yielded n = 38 and n = 128. The item-response model used eight dichotomous trigger indicators among n = 166 fear-positive participants. Boundary probabilities of 0.000 or 1.000 require caution. The broader procedural range is a class-level pattern and does not imply that most members endorsed multiple triggers.

Table S7. Item-response probabilities in the alternative three-class LCA solution

Prespecified RCT fear trigger	Class 1	Class 2	Class 3
Injection/needle fear	0.000	1.000	0.000
Fear of pain during treatment	0.458	0.228	0.849
Prolonged mouth opening	0.308	0.019	0.710
Fear of postoperative pain	0.168	0.096	0.437
Possibility of treatment failure	0.224	0.050	0.000
Sensation of endodontic instruments	0.178	0.000	0.161
Instrument sounds	0.150	0.000	0.215
Fear of postoperative swelling	0.065	0.000	0.322

Note. Estimated class proportions were 64.5%, 12.4%, and 23.2%. The pattern can be described provisionally as a lower-probability procedural class, a needle-focused class, and a broader pain/procedural class. The solution had lower AIC and higher entropy but higher BIC than the retained two-class model and is therefore presented as a plausible exploratory alternative.

Table S8. Classification quality and local-independence diagnostics for the retained two-class LCA

Diagnostic/item pair	Value/finding
Average posterior probability: needle-focused profile	0.766
Average posterior probability: procedural profile	0.998
Mean maximum posterior probability	0.945
Mean classification uncertainty	0.055
Maximum posterior probability <0.70	6.0% of participants
Bivariate residuals inspected	28 item pairs
Item pairs with BVR >3.84	6
Postoperative pain – postoperative swelling	9.298
Injection/needle – postoperative swelling	4.879
Prolonged mouth opening – postoperative pain	4.432
Pain during treatment – postoperative pain	4.399
Injection/needle – postoperative pain	4.086
Pain during treatment – instrument sounds	4.027
Item pairs with BVR >10.0	0

Note. Classification quality was high overall but uneven between classes. The six residual associations mean that local independence should be interpreted cautiously; the absence of BVR values above 10.0 is not treated as proof of negligible local dependence. Direct-association LCA models were not fitted. External comparisons used modal assignment for descriptive purposes and were not adjusted by posterior-probability weighting or a three-step procedure.

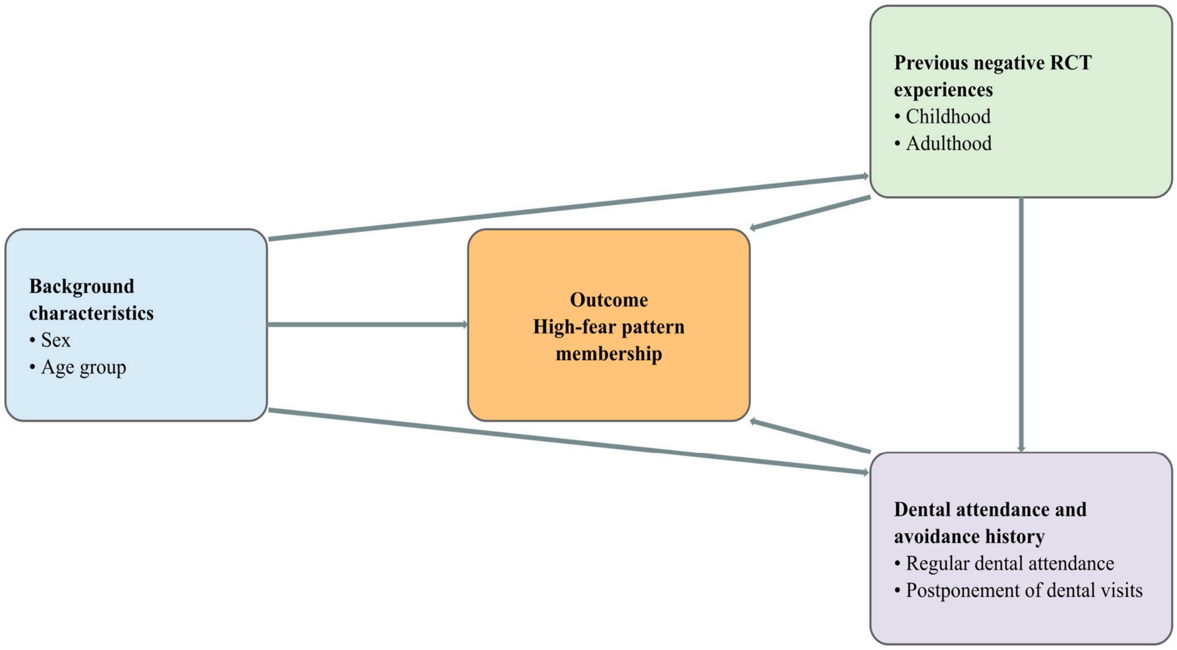

Figure S1. Conceptual directed acyclic graph (DAG) for the hierarchical association models. Arrows show prespecified hypothesized relationships used to organize predictor domains; because variables were assessed cross-sectionally, they do not establish causal effects or mediation pathways. RCT, root canal treatment.

Table S9. Fit, calibration, and stability checks for the multivariable logistic regression models

Statistic/check	Model 1	Model 2	Model 3
Analytical sample / high-fear outcomes	N = 219 / n = 67	N = 219 / n = 67	N = 219 / n = 67
Model degrees of freedom	5	8	12
Likelihood-ratio test	$\chi^2(5) = 26.792$; $p < 0.001$	$\chi^2(8) = 38.927$; $p < 0.001$	$\chi^2(12) = 50.972$; $p < 0.001$
Deviance	242.933	230.798	218.753
AIC / BIC	254.933 / 275.267	248.798 / 279.300	244.753 / 288.811
McFadden pseudo-R^2	0.099	0.144	0.189
Cox–Snell pseudo-R^2	0.115	0.163	0.208
Hosmer–Lemeshow calibration	$\chi^2(6) = 8.100$; $p = 0.231$	$\chi^2(8) = 11.690$; $p = 0.166$	$\chi^2(8) = 11.740$; $p = 0.163$
Model convergence	Converged	Converged	Converged

Note. The outcome was high-fear versus low-fear pattern membership from the revised nonredundant HCA. AIC, Akaike information criterion; BIC, Bayesian information criterion. No evidence of global complete or quasi-complete separation affecting the fitted model was observed. The 'cannot recall' category for negative RCT experience in adulthood (n = 1) yielded a non-informative boundary estimate; its category-specific AOR, 95% CI, and p-value were not reported or interpreted.

Table S10. Multicollinearity diagnostics for the hierarchical multivariable logistic regression models

Predictor	Model 1		Model 2		Model 3	
	VIF	Tolerance	VIF	Tolerance	VIF	Tolerance
Sex	1.003	0.997	1.024	0.977	1.028	0.973
Age group	1.001	0.999	1.012	0.988	1.025	0.975
Regular dental attendance			1.054	0.949	1.055	0.948
Postponement of dental visits			1.016	0.984	1.026	0.975
Negative RCT experience in childhood					1.026	0.974
Negative RCT experience in adulthood					1.039	0.963

Note. VIF, variance inflation factor. The observed VIF and tolerance values did not indicate problematic multicollinearity.
