# Supplementary material for: Profiles of Fear of Root Canal Treatment in Adult Endodontic Patients: A Cross-Sectional Study
Source: Healthcare (Basel). 2026 Aug 3;14(15):2360. doi: 10.3390/healthcare14152360 (PMC13465031; doi:10.3390/healthcare14152360)
Supplement: Supplementary file 1 [file healthcare-14-02360-s001.zip › Supplementary File S3. Completed STROBE Checklist for Cross-Sectional Studies.pdf]

Completed STROBE Checklist for Cross-Sectional Studies

Manuscript ID: healthcare-4418377

Title: Profiles of Fear of Root Canal Treatment in Adult Endodontic Patients: A Cross-Sectional Study

Item labels are concise paraphrases of the official STROBE cross-sectional checklist. Page references correspond to the 23-page tracked-change manuscript rendered for this revision. Source: https://www.strobe-statement.org/checklists/

Section	Item	Reporting requirement	Where reported
**Title / Abstract**	**1a**	Design named in title or abstract	p. 1
**Title / Abstract**	**1b**	Balanced summary of methods and findings	pp. 1–2
**Introduction**	**2**	Scientific background and rationale	pp. 2–3
**Introduction**	**3**	Objectives and hypothesis	p. 3
**Methods**	**4**	Key design elements	p. 3
**Methods**	**5**	Setting, location, and dates	p. 3
**Methods**	**6a**	Eligibility and participant selection	pp. 3–4
**Methods**	**7**	Outcomes, predictors, and external covariates	pp. 4–6
**Methods**	**8**	Data sources and measurement	pp. 4–6; Supplementary File S1
**Methods**	**9**	Efforts to address bias	p. 4; pp. 19–20
**Methods**	**10**	Basis for study size	p. 4
**Methods**	**11**	Handling and grouping of quantitative variables	p. 5
**Methods**	**12a**	Statistical methods and adjustment	pp. 5–6
**Methods**	**12b**	Subgroups and interactions	p. 6 (interaction terms not fitted); LCA subset defined
**Methods**	**12c**	Missing-data handling	p. 4 (no imputation; analysis denominators stated)
**Methods**	**12d**	Sampling strategy	p. 5 (consecutive clinic sample; no weights)
**Methods**	**12e**	Sensitivity analyses	pp. 5–6 and 11–15; Supplementary Tables S1–S6
**Results**	**13a**	Participant numbers at each stage	pp. 4 and 7

**Results**	**13b**	Reasons for exclusion or non-participation	p. 4
**Results**	**13c**	Participant flow diagram	p. 7, Figure 1
**Results**	**14a**	Participant and exposure characteristics	pp. 7–10, Figure 2
**Results**	**14b**	Missing data by variable	p. 4 (principal-analysis variables complete after exclusions)
**Results**	**15**	Outcome and summary measures	pp. 7–16
**Results**	**16a**	Unadjusted and adjusted estimates with precision	pp. 11–12; Supplementary Tables S7–S9
**Results**	**16b**	Boundaries for categorized variables	p. 5; p. 13 table note
**Results**	**16c**	Absolute risk over time, if relevant	Not applicable: cross-sectional study with no time-to-event estimand
**Results**	**17**	Other and sensitivity analyses	pp. 11–15; Supplementary Tables S1–S6
**Discussion**	**18**	Key results linked to objectives	p. 17; p. 20
**Discussion**	**19**	Limitations, bias, and imprecision	pp. 19–20
**Discussion**	**20**	Cautious interpretation and multiplicity	pp. 17–20
**Discussion**	**21**	Generalisability	pp. 19–20
**Other**	**22**	Funding and funder role	p. 21 (no external funding)
